# Potential roles of plant metabolites and Traditional Chinese Medicine formulas in regulating glycolysis-OXPHOS plasticity in gastric precancerous lesions and gastric cancer: a critical appraisal of the evidence

**DOI:** 10.3389/fphar.2026.1791005

**Published:** 2026-05-15

**Authors:** Shenghao Li, Jiao Ma, Cuicui Ma, Yanru Song, Liang Chang, Bingjie Huo

**Affiliations:** 1 Department of Integrated Traditional Chinese and Western Medicine Oncology, The Fourth Hospital of Hebei Medical University, Shijiazhuang, Hebei, China; 2 Teaching and Research Office of Basic Theories of Traditional Chinese Medicine, Hebei University of Chinese Medicine, Shijiazhuang, Hebei, China

**Keywords:** gastric cancer, gastric precancerous lesions, glycolysis, oxidative phosphorylation, plant metabolites, traditional Chinese medicine formulas

## Abstract

The Warburg effect in tumor cells is characterized by increased glucose uptake, enhanced glycolytic flux, and the conversion of pyruvate to lactate for energy generation, rather than its utilization in oxidative phosphorylation (OXPHOS), even under aerobic conditions. This metabolic reprogramming, coupled with OXPHOS dysfunction, is closely correlated with the initiation, invasion, metastasis, chemoresistance, and poor prognosis of gastric precancerous lesions (GPL) and gastric cancer (GC). Therefore, modulating the glycolytic and OXPHOS-related signaling pathways intertwined with microbial regulation presents a novel therapeutic strategy for GPL and GC treatment. Plant metabolites and TCM formulas exhibit potential advantages in regulating GPL and GC metabolism because they may modulate multiple steps of glycolysis and influence OXPHOS-related abnormalities in preclinical models, thereby contributing to restoration of mitochondrial functional homeostasis. However, these effects should generally be interpreted as metabolic modulation observed in experimental systems rather than definitive evidence of direct molecular targeting. We found that botanical drugs and plant metabolites were frequently associated with changes in GPL and GC metabolism through signaling networks involving phosphatidylinositol 3-kinase (PI3K)/protein kinase B (AKT), Hippo, hypoxia-inducible factor (HIF)-1α, c-Myc, and non-coding RNAs. These regulatory effects were accompanied by modulation of glycolysis- and OXPHOS-related enzymes and markers, and were often linked to reduced proliferation, invasion, metastasis, and chemoresistance in GPL and GC models. Nevertheless, for many pathways, the available evidence remains correlative rather than demonstrating direct target engagement. Plant metabolites and TCM formulas exhibit distinct advantages in regulating GPL and GC metabolism, as they can modulate multiple steps of glycolysis and modulate dysregulated OXPHOS pathways in GPL and GC cells to restore mitochondrial functional homeostasis. This process involves the transient modulation of specific OXPHOS complexes to correct abnormal mitochondrial energy metabolism and achieve the net restoration of ATP balance. Leveraging plant metabolites and TCM formulas to modulate these metabolic processes thus represents a promising therapeutic approach for GPL and GC management. This review summarized the research progress on the intervention effects of botanical drugs and plant metabolites on GPL and GC metabolic reprogramming, based on relevant studies retrieved from Chinese databases (CNKI, Wanfang, VIP) and English databases (PubMed, Web of Science, Embase) from the establishment of each database to October 2025, using a combination of subject terms and free words (including “gastric cancer, gastric precancerous lesions, glycolysis, oxidative phosphorylation, OXPHOS, botanical drug, plant metabolite, TCM formula” and their corresponding Chinese translations) restricted to the title, abstract, and keyword fields. We found that botanical drugs and plant metabolites primarily regulated GPL and GC metabolism by modulating key signaling pathways, including phosphatidylinositol 3-kinase (PI3K)/protein kinase B (AKT), Hippo, hypoxia-inducible factor (HIF)-1α, c-Myc, and non-coding RNAs. These regulatory effects further modulated the activity of enzymes associated with glycolysis and OXPHOS, ultimately inhibiting the proliferation, invasion, and metastasis of GPL and GC cells while enhancing their chemosensitivity. Elucidating the regulatory mechanisms of botanical drugs and plant metabolites on GPL and GC metabolic reprogramming, including glycolysis and OXPHOS, not only helped clarify the therapeutic basis of plant metabolites and TCM formulas against GPL and GC but also provided a theoretical foundation for the development of novel anti-GC strategies.

## 1 Introduction

In 2022, 968,350 people were diagnosed with gastric cancer (GC) worldwide, with 659,853 deaths from the disease, making GC the fifth most common cancer and the fifth leading cause of cancer-related deaths globally (Bray et al., 2024). The majority of GC cases worldwide were believed to stem from *Helicobacter pylori* (Hp) infection and Hp-induced carcinogenic progression (Plummer et al., 2015; de Martel et al., 2020). Chronic inflammatory damage induced by Hp colonization triggers a stepwise sequence of precancerous lesions of increasing severity, progressing from non-atrophic gastritis to atrophic gastritis, intestinal metaplasia, dysplasia, and ultimately invasive cancer (Huang et al., 2023). These precursors, collectively referred to as gastric precancerous lesions (GPL), are critical intermediate stages in GC carcinogenesis, and their metabolic disorders often precede malignant transformation. Additionally, metabolic disorders in the tumor microenvironment are decisive factors in tumor formation and underlie gastrointestinal carcinogenesis. key proteins involved in glucose metabolism and metabolic intermediates undergo profound alterations during the malignant transition from GPL to GC (Li X. et al., 2025). In the 1920s, Otto Warburg first demonstrated that unlike normal cells, tumor cells predominantly convert glucose to lactate even under aerobic conditions, a phenomenon termed aerobic-condition glycolysis or the Warburg effect, characterized by augmented glucose uptake and lactate production (Warburg, 1956). Notably, accumulating evidence indicates that Warburg effect-related metabolic aberrations are already established in advanced GPLs, thereby laying a functional foundation for subsequent malignant progression. It is critical to clarify that mitochondria do not directly catabolize glucose; rather, glucose is first metabolized to pyruvate in the cytosol, with pyruvate serving as the primary substrate for mitochondrial oxidative phosphorylation (OXPHOS) (Vander Heiden et al., 2009). Tumor metabolic heterogeneity, a hallmark feature of GC, dictates the dynamic balance between the Warburg effect and OXPHOS. GC mitochondria exhibit structural and functional impairments, including mtDNA mutations and compromised respiratory chain activity, which collectively modulate the Warburg effect: these defects reduce OXPHOS efficiency, forcing GC cells to rely more heavily on glycolysis and thereby exacerbating the Warburg phenotype (Arjunan et al., 2025). Similar mitochondrial dysfunction has been documented in GPL, contributing to their metabolic reprogramming and progressive transformation. However, the precise nature of OXPHOS dysfunction in both GPL and GC remains incompletely defined in current literature, and the role of OXPHOS modulators in governing metabolic reprogramming across these pathological states represents a critical unresolved question in the field.

Originating from gastric epithelial cells, GC, similar to other tumor types, exhibits the Warburg effect, including increased glucose uptake, enhanced glycolysis, and conversion of substantial pyruvate to lactate rather than OXPHOS to provide energy under aerobic conditions (Warburg, 1956). Aerobic-condition glycolysis serves as a pivotal metabolic node underlying tumor cell proliferation, growth, and invasion, as well as the malignant progression of GPL. Elucidating the mechanisms underlying glucose metabolic reprogramming in both GC and GPL is therefore imperative for uncovering the fundamental drivers of GC development and identifying novel therapeutic targets. It should be noted that simply inhibiting glycolysis is not always enough to eradicate GC cells, as they can typically switch to rely on OXPHOS in an attempt to overcome the metabolic insult induced by glycolysis inhibition.

Plant metabolites and TCM formulas have traditionally served as primary therapeutic agents in various traditional medical systems and remain important resources for modern drug discovers. Many plant metabolites and TCM formulas possess anti-cancer properties. Studies have shown that β-asarone, the main active metabolite of the botanical drugs *Rhizoma Acori Tatarinowii*, enhances chemosensitivity by inhibiting tumor glycolysis in GC (Tao et al., 2020), though its potential carcinogenic risks and toxicological profiles require careful evaluation. Gypenoside, a widely used plant metabolite, inhibited GC proliferation by inhibiting glycolysis through the Hippo pathway (Pan et al., 2024).

Thus, investigating plant metabolites and TCM formulas that exhibit both antiglycolytic activity and potential OXPHOS-regulatory effects holds significant promise for developing novel therapeutic strategies for GPL and GC. This review aimed to elucidate the glycolytic mechanisms in GC cells, clarified the characteristics of OXPHOS dysfunction in GC, and summarized plant metabolites and TCM formulas with potential antiglycolytic properties and OXPHOS-modulating effects, thereby facilitating the development and clinical translation of novel anti-GC therapeutics and precancerous intervention strategies.

To conduct a comprehensive review of plant metabolites and TCM formulas modulating glycolysis-OXPHOS plasticity in GPL and GC, relevant studies were retrieved from Chinese databases (CNKI, Wanfang, VIP) and English databases (PubMed, Web of Science, Embase) spanning from the establishment of each database to October 2025. A combination of subject terms and free words was used for retrieval, including “gastric cancer, gastric precancerous lesions, glycolysis, oxidative phosphorylation, OXPHOS, botanical drug, plant metabolite, TCM formula” and their corresponding Chinese translations. The search was restricted to the title, abstract, and keyword fields. Included studies were required to report clear mechanistic evidence regarding the regulatory effects of plant metabolites or TCM formulas on glycolysis and/or OXPHOS in GC and/or GPLs. Conversely, duplicate publications, conference abstracts only, and studies with flawed experimental designs were excluded. Finally, after independent study selection and resolution of discrepancies by two independent reviewers, eligible references were included in the final systematic review.

## 2 Role of glycolysis in gastric precancerous lesions and gastric cancer

The hexokinase (HK) protein family catalyzes the conversion of glucose to glucose-6-phosphate (G6P), which is the first rate-limiting step in glycolysis. This enzyme family consists of four major subtypes, namely HK1, HK2, HK3, and HK4 (Lis et al., 2016). Among these subtypes, HK2 expression is significantly upregulated in GC and is closely associated with the enhancement of aerobic-condition glycolysis (Rho et al., 2007).

Pyruvate kinase (PK) is a key enzyme involved in the final step of glycolysis, which catalyzes the conversion of phosphoenolpyruvate to pyruvate. The PK protein family consists of four members: PKM1, PKM2, PKL, and PKR (Dong et al., 2016). Among these isoforms, PKM2 exhibits significantly elevated expression levels in multiple cancer cells and is closely associated with poor clinical prognosis (Shang et al., 2016). Specifically, PKM2 expression was notably increased in GC tissues and is significantly associated with lymph node metastasis and advanced TNM staging (Gao et al., 2015). Mechanistically, PKM2 promoted cell migration, inhibited autophagy, and facilitated the malignant progression of GC by mediating the activation of the phosphatidylinositol 3-kinase/protein kinase B (PI3K/AKT) pathway (Wang C. et al., 2017).

The regulation of glycolysis is also influenced by glucose transporters (GLUTs). Among the 14 members of the GLUT family, GLUT1 through GLUT5 have been the most intensively studied to date. These transporters function as glucose and/or fructose carriers in a variety of tissues and cell types (Pyla et al., 2013; Cui et al., 2022). Notably, suppression of GLUT1 expression reversed the Warburg effect and induced apoptosis in MKN45 cells (Zhang et al., 2015).

Lactate dehydrogenase (LDH), the enzyme that catalyzes the irreversible conversion of pyruvate to lactate, plays a critical role in the metabolic reprogramming of tumors (Sharma et al., 2022). LDH-A expression was an independent prognostic risk factor for GC patients, and reducing LDHA levels inhibited cell migration and invasion (Zhu et al., 2018).

**FIGURE 1:**
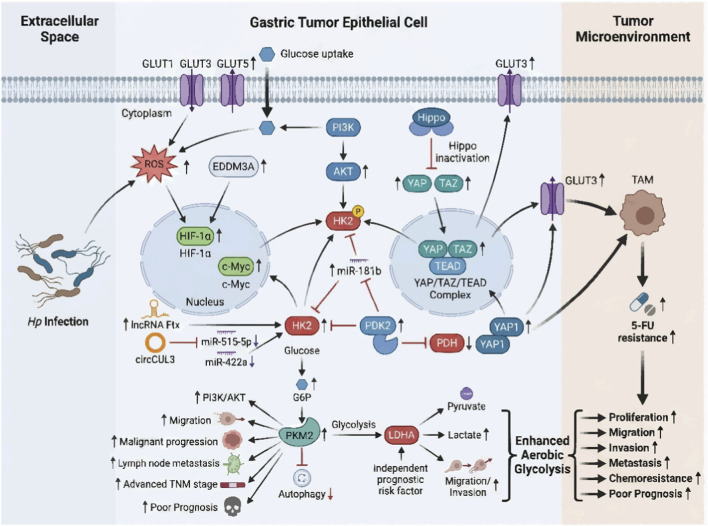
Schematic representation of the key glycolytic regulators and their interconnected signaling networks in gastric cancer. This diagram illustrates the core glycolytic enzymes (HK2, PKM2, LDHA), upstream drivers (Hp infection, ROS, HIF-1α, c-Myc, PI3K/AKT, Hippo/YAP), and non-coding RNA modulators (lncRNA Ftx, circCUL3, miR-422a, miR-181b) that collectively orchestrate enhanced aerobic-condition glycolysis. This metabolic reprogramming drives malignant phenotypes including proliferation, migration, invasion, metastasis, and chemoresistance in gastric cancer.

Numerous studies have confirmed that the development and progression of GC are associated with glycolysis, involving multiple molecular mechanisms. These complex regulatory networks are illustrated in Figure 1. Hp infection is widely recognized as one of the key contributing factors in gastric carcinogenesis. This infection markedly increases the levels of reactive oxygen species (ROS) in GC cells, which in turn upregulates hypoxia-inducible factor (HIF)-1α expression and facilitates the glycolytic process in GC (Bhattacharyya et al., 2010). EDDM3A has been demonstrated to drive GC progression by facilitating HIF-1α-dependent aerobic-condition glycolysis the progression of GC by promoting HIF-1α-dependent aerobic-condition glycolysis (Wu T. et al., 2022). Meanwhile, the oncogene c-Myc promoted glycolysis by upregulating the expression of several key glycolytic enzymes. Knocking out of c-Myc in GC cells significantly suppressed cell proliferation and glycolytic activity (Gao et al., 2019). Several non-coding RNAs have also been implicated in regulating aerobic-condition glycolysis in GC cells. In GC cells, LncRNA Ftx has been found to promote HK2 expression (Zhu et al., 2020), while circCUL3 promotes glycolysis in GC cells by modulating the miR-515–5p/HK2 axis (Pu et al., 2020). miR-422a was downregulated in GC. It inhibited PDK2 and restored the activity of PDH, leading to an increased Warburg effect in GC cells (He et al., 2018). MiR-181b reduced glucose uptake and increases ATP production by inhibiting HK2 expression (Li et al., 2016). The PI3K/AKT pathway is a crucial signaling cascade regulating glucose metabolism. AKT phosphorylates and activates HK2 by enhancing its mitochondrial integration (Roberts et al., 2013; Harvey et al., 2003; Justice et al., 1995). When Hippo signaling is inactivated, YAP/WW domain-containing transcription regulator 1 (TAZ) translocate to the nucleus and bind to transcription enhancer factor domain (TEAD) transcription factors, inducing the expression of oncogenic genes (Park et al., 2020). The YAP agonist XMU-MP-1 promoted the proliferation of GC cells and the glycolytic process (Pan et al., 2024). In addition, YAP1 promoted 5-fluorouracil (5-FU) resistance in GC by regulating GLUT3-dependent metabolic reprogramming in tumor-associated macrophages (He et al., 2021).

## 3 Modulation of glycolysis in gastric precancerous lesions and gastric cancer

**FIGURE 2:**
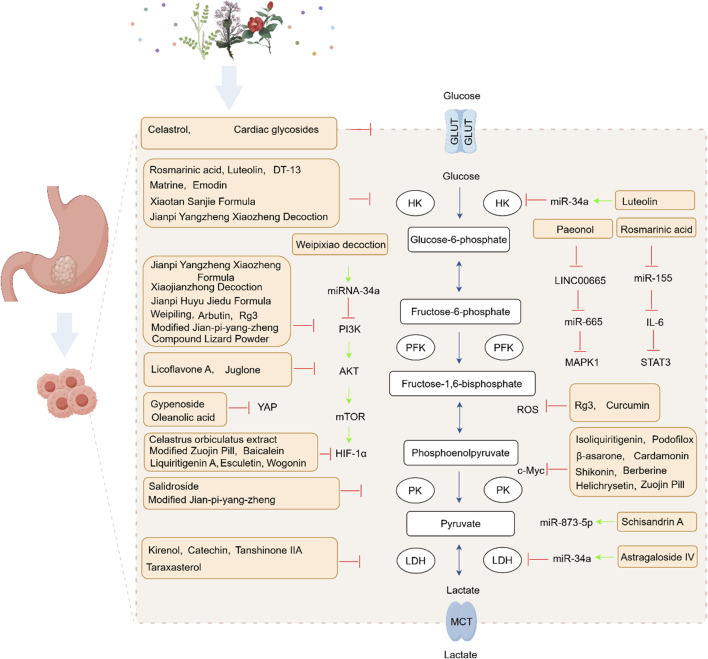
Plant metabolites and traditional Chinese medicine formulas and modulating the glycolysis in gastric cancer.

Based on the available preclinical literature, plant metabolites and TCM formulas may modulate glucose metabolic reprogramming in GPL and GC predominantly through glycolysis-related mechanisms. Accordingly, this review mainly summarizes studies addressing glycolysis-related regulation (Figure 2; Tables 1, 2). The reported effects of these interventions are commonly associated with changes in glycolytic enzymes and signaling markers, including PI3K/Akt, Hippo, non-coding RNAs (ncRNAs), c-Myc, HIF-1α, ROS, the metabolism-immunity axis, and RTKN. However, in most included studies, these observations should be interpreted as pathway-associated metabolic modulation rather than definitive evidence of direct molecular targeting.

**TABLE 1:** Effects of plant metabolites modulating glycolysis on gastric precancerous lesions and gastric cancer.

Plant metabolites	Experimental model	Mechanism	References
DT-13	*In vitro*: BGC-823, MYH-9 knockdown cells; *in vivo*: subcutaneous/orthotopic xenograft models in nude mice Dose/concentration range: BGC-823 cells and MYH-9 knockdown cells were treated with DT-13 at 10 μM combined with topotecan (TPT) at 0.1–1 μM for 48 h; BALB/c nude mice bearing subcutaneous/orthotopic xenografts were treated with DT-13 at 0.625–2.5 mg/kg via intragastric administration once daily, combined with TPT at 0.5 mg/kg via intravenous injection twice weekly for the relevant period; the vehicle group was given 0.5% CMC-Na synchronously for intragastric administration and equal volume of normal saline synchronously for intravenous injection	Reported to inhibit tumor growth, suppress glycolytic enzyme activity, and reduce HK2 binding to mitochondria	Yu et al. (2019)
Emodin	*In vitro*: GES-1, MGC803 cells Dose/concentration range: GES-1 cells were treated with emodin at 0, 25, 50, 75, 100, 125 μmol/L; MGC803 cells were treated with emodin at 0, 5, 10, 20, 30, 60 μmol/L. Cells were cultured for 24/48 h, respectively	Inhibits the key glycolytic enzyme HK2 and downregulate the oncogene FOXD1	Liu S. et al. (2025)
Luteolin	*In vitro*: AGS cells Dose/concentration range: AGS cells were treated with luteolin at 20, 40, 80 μmol/L	Inhibits aerobic-condition glycolysis by down-regulating the expression of HK2, PKM2 and GLUT1, and induces apoptosis of AGS cells	Shang et al. (2023)
Rosmarinic acid	*In vivo*: BALB/c-nu/nu nude mice with MKN-45 gastric cancer xenografts Dose/concentration range: BALB/c (nu/nu) nude mice bearing human gastric cancer MKN-45 cells were treated with rosmarinic acid at 1 mg/kg, 2 mg/kg, and 4 mg/kg via intraperitoneal injection of 0.2 mL each time, twice daily for 18 consecutive days. The control group was given an equal volume of normal saline via intraperitoneal injection	Inhibits the Warburg effect by down-regulating the expression of HK2 and LDHA, reducing glucose consumption and lactate production, and suppresses tumor growth by inhibiting the IL-6/STAT3/HIF-1α signaling pathway	Wei et al. (2011)
Matrine	*In vitro*: HGC-27 cells Dose/concentration range: HGC-27 cells were treated with matrine at 5, 10, 20 μmol/L	Inhibits the expression of glycolytic key enzymes HK, PDH and CS, reduces lactate production and inhibits the migration and invasion of HGC-27 cells	Zeng and Lu (2021)
Salidroside	*In vitro*: SGC-7901 and MKN-45 cells; *in vivo*: BALB/c nude mice xenografted with SGC-7901 and MKN-45 cells Dose/concentration range: SGC-7901 and MKN-45 cells were treated with Salidroside at 0,10,20,40,80 μM for 24 h; the xenograft model mice were treated with Salidroside at 20 mg/kg via intraperitoneal injection once daily from day 10 to day 17 after inoculation	Inhibits the expression of glycolysis-related enzymes ENO1 and PKM2 and GLUT1 and induces apoptosis of gastric cancer cells	Dai et al. (2021)
Kirenol	MNU-induced gastric cancer rat model Dose/concentration range: Mice were treated with MNU at 100 mg/kg via intragastric administration thrice weekly combined with Kirenol at 30 mg/kg via intragastric administration once daily for 16 consecutive weeks; the 5-FU positive control group was treated with MNU at 100 mg/kg via intragastric administration thrice weekly combined with 5-FU at 40 mg/kg via intraperitoneal injection once weekly for 16 consecutive weeks; the control group was given citrate buffer plus 5% saline via intragastric administration thrice weekly for 16 consecutive weeks	Inhibits the status of serum markers of GC and gastrin, ALP, LDH, and γ-GT.	Liu et al. (2020a)
Catechin	*In vitro*: SNU620, SNU620/5FU, and AGS cells Dose/concentration range: SNU620, SNU620/5FU, and AGS cells were treated with catechin at 0, 5, 10, 25,50 μmol/L for 48 h	Decreases LDHA activity and lactate production	Han et al. (2021)
Tanshinone IIA	*In vitro*: MGC803 cells Dose/concentration range: MGC803 cells were treated with Tanshinone IIA at 0, 6.25, 12.5, 25, 50, 100, 200 μmol/L for 24 h and 48 h	Inhibits the proliferation of MGC803 cells and reduces the expression of LDHA and PFKP	Liu Z. et al. (2025)
Taraxasterol	*In vitro*: HGC-27, GES-1 cells Dose/concentration range: HGC-27 and GES-1 cells were treated with taraxasterol at 0, 5, 10, 15, 20 μmol/L for 48 h	Suppresses the proliferation of HGC-27 cells and promotes apoptosis by inhibiting glycolysis	Zhao et al. (2021)
Cardiac glycosides	*In vitro*: MKN45 cells Dose/concentration range: MKN45 cells were treated with Cardiac glycosides at 0.2 μmol/L for 24 h	Decreases the expression of GLUT1	Fujii et al. (2022)
Celastrol	*In vitro*: BGC-823 cells Dose/concentration range: BGC-823 cells were treated with celastrol at 0, 0.75, 1.0, 1.25, 1.5, 1.75, 2.0 μmol/L	Reduces cellular glucose utilization and lactate production, and inhibits the activities of GLUT1, HK2, and LDH	Li K. et al. (2019)
Licoflavone A	*In vitro*: MKN45 and SGC7901 cells; *in vivo*: MKN45 xenograft mouse model Dose/concentration range: MKN45 and SGC7901 cells were treated with Licochalcone A at 0, 15, 30, 60 μmol/L for 12/24/48/72 h; GES-1 cells were treated with Licochalcone A at 0, 7.5, 15, 30, 60, 120, 180 μmol/L for 72 h. BALB/c nude mice bearing MKN45 xenografts were treated with Licochalcone A at 10 mg/kg via intraperitoneal injection (once daily)	Suppresses tumor glycolysis in gastric cancer cells by down-regulating the expression of HK2	Wu et al. (2018)
Juglone	*In vitro*: SGC-7901 cells Dose/concentration range: SGC-7901 cells were treated with Juglone at 0, 1, 5, 10 μmol/L	Inhibits the activity of AKT/mTOR signaling pathway	Yu and Jin (2022)
Arbutin	*In vitro*: SNU-601 cells Dose/concentration range: SNU-601 cells were treated with Arbutin at 0, 12.5, 25, 50, 100, 200, 400 μmol/L	Inhibits the malignant progression of gastric cancer cells by suppressing the PI3K/Akt/GLUT1 signaling pathway	Han et al. (2024)
Compound Lizard Powder	*In vitro*: MKN45 and MKN45/DDP (cisplatin-resistant) cells; *in vivo*: BALB/c nude mouse model with MKN45/DDP xenograft tumors Dose/concentration range: Human gastric cancer MKN45 and MKN45/DDP cells were treated with cisplatin at 0.1, 0.2, 0.4, 0.8, 1.6, 3.2 μg/mL for 48 h to detect drug resistance. BALB/c nude mice bearing MKN45/DDP xenografts were randomly divided into four groups: model group (normal saline, intragastric gavage), cisplatin group (0.002 g/kg, intraperitoneal injection, twice a week), Compound Lizard Powder group (2.8 g/kg, intragastric gavage, twice a day), and combination group (the same doses as single drug groups)	Suppresses the activation of the PI3K/Akt signaling pathway	Cheng et al. (2024)
Gypenoside	*In vitro*: HGC-27 and AGS cells; *in vivo*: BALB/c nude mouse model with HGC-27 xenograft tumors Dose/concentration range: Human gastric cancer HGC-27 and AGS cells were treated with gypenoside at 0, 50, 75, 100, 125, 150 μM for 48 h; BALB/c nude mice bearing HGC-27 xenografts were intraperitoneally injected with gypenoside at 30 mg/kg once daily for 14 consecutive days after 7 days of cell inoculation	Suppresses gastric cancer cell proliferation and migration by inhibiting glycolysis	Pan et al. (2024)
Oleanolic acid	*In vitro*: MKN-45 and SGC-7901 cells Dose/concentration range: Human gastric cancer MKN-45 and SGC-7901 cells were treated with oleanolic acid at 0, 10, 20, 30, 40, 50, 60, 70, 80 μM for 24 h	Reduces the viability and proliferation of gastric cancer cells by inhibiting aerobic-condition glycolysis	Li Y. et al. (2019)
Ginsenoside Rg3	*In vivo*: ATP4a^−/−^ mouse model Dose/concentration range: Atp4a^−/−^ mice (gastric precancerous lesion, GPL model) were randomly divided into model group, low-dose Rg3 group (5 mg/kg), and high-dose Rg3 group (10 mg/kg); wild-type C57Bl/6 mice served as the control group. All groups were treated via intragastric gavage once daily for 10 consecutive weeks	Decreases the expression of miRNA-21	Liu et al. (2020b)
Astragaloside IV	*In vivo*: MNNG-induced PLGC rats Dose/concentration range: SD rats were randomly divided into normal group, model group, low-dose astragaloside IV group (50 mg/kg), and high-dose astragaloside IV group (100 mg/kg). The model group was induced with gastric precancerous lesions by *ad libitum* drinking of 200 mg/L MNNG solution and alternate-day feeding for 16 weeks. After successful modeling, the astragaloside IV groups were treated via intragastric gavage once daily for 10 consecutive weeks; the normal group and model group were given equal volume of distilled water synchronously	Increases the expression of miRNA-34a and TIGAR	Zhang C. et al. (2018)
Rosmarinic acid	*In vitro*: MKN45 cells; *in vivo*: BALB/c nude mouse model with MKN45 xenograft tumors Dose/concentration range: MKN-45 cells were treated with Rosmarinic acid at 0–700 μM for 24 h. BALB/c-nu mice were treated with Rosmarinic acid at 2 mg/kg via intraperitoneal injection once daily for 14 consecutive days; the control group was given equal volume of normal saline synchronously	Decreases the expression of miRNA-155–5p	Han et al. (2015)
Paeonol	*In vitro*: Apatinib-resistant gastric cancer cell lines (BGC-823/AP and MGC-803/AP); *in vivo*: BALB/c nude mouse model with BGC-823/AP cell-derived lung metastasis Dose/concentration range: BGC-823/AP and MGC-803/AP cells were treated with paeonol at 0,20,40,60,80,100,120 mg/L for 24/48/72h; BALB/c nude mice were treated with paeonol at 0,30,50 mg/kg via intraperitoneal injection once daily for 28 consecutive days; the control group was given equal volume of the corresponding solvent synchronously	Downregulates LINC00665 expression, upregulates miR-665 expression, and downregulates MAPK1 expression	Li et al. (2022)
Schizandrin A	*In vitro*: SGC-7901 cells Dose/concentration range: SGC-7901 cells were treated with Schisandrin A at 50μM and 100 μM for 48 h; the cells in the combined intervention groups were treated with Schisandrin A at 100 μM and transfected with corresponding plasmids for 48 h	Upregulates miR-873–5p expression	Wang Z. et al. (2024)
Isoliquiritigenin	*In vitro*: GES-1, MGC803 and SGC7901 cells; *in vivo*: nude mouse model with MGC803 cell xenografts Dose/concentration range: GES-1, MGC803 and SGC7901 cells were treated with Isoliquiritigenin at 5–1280 μM for 24–72h; MGC803 cells were treated with Isoliquiritigenin at 20,40,60 μM for 24h and SGC7901 cells were treated with Isoliquiritigenin at 30,50,70 μM for 24h for apoptosis detection; MGC803 cells were treated with Isoliquiritigenin at 40 μM and SGC7901 cells were treated with Isoliquiritigenin at 50 μM for 12,16,20,24h for metabolism-related detection. BALB/c nude mice were treated with Isoliquiritigenin at 10,30,100 mg/kg via gavage once daily for 21 consecutive days; the model group was given equal volume of normal saline synchronously	Induces PDHK1/PGC-1α-mediated energy metabolic collapse by depressing the protein expression of c-Myc and HIF-1α	Yu et al. (2023)
Podofilox	*In vitro*: AGS and HGC-27 cells Dose/concentration range: AGS and HGC-27 cells were treated with Podofilox at 0, 0.3125, 0.625, 1.25, 2.5, 5, 10, 20, 40, 80 nM for 48 h for IC_50_ detection; the 2 cell lines were treated with Podofilox at 3.4 nM for 1–5 days for cell viability detection, for 7 days for colony formation detection, and for 48 h for cell cycle and protein expression detection	Suppresses gastric cancer cell proliferation by regulating cell cycle arrest and the c-Myc/ATG10 axis	An et al. (2021)
β-asarone	*In vitro*: MGC803, SGC7901, MKN74 cells Dose/concentration range: MGC803, SGC7901 and MKN74 cells were treated with β-Asarone at 0,7.5,15,30,60,120 μg/ml for 24 h	Downregulates expression of HIF-1α and c-Myc	Tao et al. (2020)
Cardamonin	*In vitro*: MGC-803, SGC-7901 cells; *in vivo*: BALB/c nude mouse model with MGC-803 xenograft tumors Dose/concentration range: MGC-803 and SGC-7901 cells were treated with Cardamonin at 1–200 μM for 24 h	Blocks the c-Myc/GLUT4 axis to inhibit energy metabolic reprogramming	Liang (2021)
Shikonin	*In vitro*: SGC-7901, GES-1 cells Dose/concentration range: GES-1 and SGC-7901 cells were treated with Shikonin at 2,5,10,20,40 μM for 24 h	Downregulates expression of c-Myc protein	Zhang J. X. et al. (2023)
Berberine	*In vitro*: MKN45 cells Dose/concentration range: MKN45 cells were treated with Berberine at 0,6,12,24,48,96 μmol/L for 48 h	Downregulates the expression of β-catenin and c-Myc proteins	Zhang et al. (2020)
Helichrysetin	*In vitro*: MGC803, SGC7901 cells; *in vivo*: BALB/c nude mouse model with MGC803 xenograft tumors Dose/concentration range: MGC803 and SGC7901 cells were treated with Helichrysetin at 5,10,20,40,80,120,160,200 μM for 48 h; BALB/c nude mice bearing MGC803 cell xenografts were treated with HEL at 3,10,30 mg/kg via intraperitoneal injection once daily for 21 consecutive days; the positive control group was treated with 5-FU at 50 mg/kg via intraperitoneal injection twice a week for 21 consecutive days; the control group was given equal volume of normal saline via intraperitoneal injection synchronously	Suppresses c-Myc expression and transcriptional activity, downregulates PDHK1 and LDHA expression	Wang et al. (2022)
Celastrus orbiculatus	*In vivo*: MNNG-induced PLGC rats Dose/concentration range: SD rats with precancerous lesions of gastric cancer induced by MNNG were treated with Celastrus orbiculatus extract at 12.5,25,50 mg/kg via gavage once daily for 4 consecutive weeks; the normal control group was given equal volume of normal saline via gavage synchronously, and the model group was not given Celastrus orbiculatus intervention	Downregulates protein and mRNA expression of HK2, PKM2, GLUT1, LDHA and HIF-1α	Zhang X. Z. et al. (2023)
Baicalein	*In vitro*: AGS cells Dose/concentration range: AGS cells were treated with Baicalein at 0,10,20,40,60,80 μM for 24/48 h	Downregulates the expression of HIF-1α, HK2 and LDHA	Chen et al. (2015)
Liquiritigenin A	*In vitro*: AGS cells Dose/concentration range: AGS cells were cultured under hypoxic conditions (5% O_2_, 5% CO_2_, 90% N_2_) and divided into hypoxia group, Licoflavone A low (25 μmol/L), medium (50 μmol/L), high (100 μmol/L) concentration groups, with a normoxia group (5% CO_2_) as control; the cells were treated with corresponding concentrations of Licoflavone A for 24/48 h	Suppresses glycolysis by inhibiting the HIF-1α signaling pathway	Dong et al. (2024)
Aesculetin	*In vitro*: SGC-7901 cells Dose/concentration range: SGC-7901 cells were treated with Aesculetin at 0.05,0.072,0.096,0.10,0.144,0.20,0.40,0.80 mmol/L for 48 h	Suppresses glycolysis by inhibiting the HIF-1α signaling pathway	Jia et al. (2024)
Wogonin	*In vitro*: SGC-7901 cells Dose/concentration range: SGC-7901 cells were treated with Wogonin at 5,10,15,20,25,30 μg/mL for 48 h	Suppresses glycolysis by inhibiting the HIF-1α signaling pathway	Wang et al. (2019)
Ginsenoside Rg3	*In vivo*: MNNG-induced GPL rats Dose/concentration range: SD rats with gastric precancerous lesions induced by MNNG combined with ammonia were treated with Ginsenoside Rg3 at 1.8,3.6,7.2 mg/kg via gavage once daily for 12 consecutive weeks; the positive control group was treated with folic acid at 2.7 mg/kg via gavage synchronously, and the normal control group and model group were given equal volume of normal saline via gavage synchronously	Increases intracellular ROS concentration	Lv et al. (2022)
Curcumin	*In vivo*: SGC-7901, BGC-823 cells; *in vivo*: nude mouse model with BGC-823 cells xenograft tumors Dose/concentration range: SGC-7901 and BGC-823 human gastric cancer cells were treated with curcumin micelles or native curcumin at 5,10,20,30,40 μg/mL for 24 h	Increases intracellular ROS concentration	Lin et al. (2020)
GO-Y022	*In vitro*: SH-10-TC, GCIY cells; *in vivo*: K19-Wnt1/C2 mE (Gan) mouse model of gastric cancer Dose/concentration range: SH-10-TC and GCIY human gastric cancer cells were treated with GO-Y022 (pyrolyzed deketene curcumin) at 5 mM alone or combined with 2-deoxy-D-glucose (2DG) at 5 mM for 3h; K19-Wnt1/C2 mE (Gan) mice with gastric cancer were fed a high-fat diet containing 0.5% (w/w) GO-Y022 daily for 7 consecutive weeks; the control group was fed a high-fat diet alone	Reduces L-lactate production and blocks Treg generation	MaruYama et al. (2023)

**TABLE 2:** Effects of TCM formulas modulating glycolysis on gastric precancerous lesions and gastric cancer.

Name	Composition (Chinese, English, Latin names)	Experimental model	Mechanism	References
Jianpi Yangzheng Xiaozheng Decoction	Dangshen (Codonopsis Root, Codonopsis pilosula (Franch.) Nannf.), Huangqi (Milkvetch Root, Astragalus membranaceus (Fisch.) Bunge), Fuchao Baizhu (Bran-fried Atractylodes Rhizome, Atractylodes macrocephala Koidz.), Chao Baishao (Stir-fried White Peony Root, Paeonia lactiflora Pall.), Danggui (Chinese Angelica Root, Angelica sinensis (Oliv.) Diels), Shijianchuan (Chinese Sage Herb, Salvia chinensis Benth.), Sanleng (Common Burreed Tuber, Sparganium stoloniferum (Buch.-Ham. ex D.Don) Buch.-Ham.), Ezhu (Zedoary Rhizome, Curcuma phaeocaulis Val.), Baihua Sheshecao (Hedyotis Herb, Hedyotis diffusa Willd.), Chenpi (Dried Tangerine Peel, Citrus reticulata Blanco), Gancao (Licorice Root, Glycyrrhiza uralensis Fisch.)	*In vitro*: HGC-27, AGS cells Dose/concentration range: HGC-27 and AGS cells were assigned to drug-containing serum group (79% RPMI-1640 + 20% rat drug-containing serum+1% penicillin-streptomycin) and control serum group (79% RPMI-1640 + 20% rat control serum+1% penicillin-streptomycin). Cells were cultured in serum-free medium for 2 h prior to treatment	Associated with reduced HK2 expression, decreased glucose uptake and lactate production, and increased apoptosis in GC models	Yang (2016)
Xiaotan Sanjie Formula	Zhinanxing (Prepared Arisaema Rhizome, Arisaema erubescens (Wall.) Schott), Zhibanxia (Prepared Pinellia Tuber, Pinellia ternata (Thunb.) Breit.), Quanxie (Scorpion, Buthus martensii Karsch), Wugong (Centipede, Scolopendra subspinipes mutilans L. Koch), Fuling (Poria Cocos, Poria cocos (Schw.) Wolf), Gancao (Licorice Root, Glycyrrhiza uralensis Fisch.)	*In vivo*: BALB/c-nu/nu mice with MNK-45 human gastric cancer xenografts Dose/concentration range: BALB/c-nu/nu mice bearing MKN-45 human gastric cancer xenografts were treated with Xiaotan Sanjie decoction at 0.4 mL per mouse per day via gavage for 6 consecutive weeks; the positive control group was treated with capecitabine suspension at 0.4 mL per mouse per day (267 mg/kg) via gavage with 2 consecutive weeks of administration followed by a 1-week withdrawal interval for the whole 6-week period; the control group was given equal volume of normal saline via gavage once daily for 6 consecutive weeks	Associated with reduced HK2 expression in serum and tumor tissues, lower glycolysis-related readouts, and suppressed tumor growth in xenograft models	Ruan et al. (2021)
Modified Jian-pi-yang-zheng	Dangshen (Codonopsis Root, Codonopsis pilosula (Franch.) Nannf.), Huangqi (Milkvetch Root, Astragalus mongholicus Bunge), Sanleng (Common Burreed Tuber, Sparganium stoloniferum (Buch.-Ham. ex D.Don) Buch.-Ham.), Ezhu (Zedoary Rhizome, Curcuma phaeocaulis Val.)	*In vitro*: MGC-803, SGC-7901 and BGC-823 cells; *in vivo*: BALB/c nude mice xenografted with SGC-7901 cells Dose/concentration range: MGC-803, SGC-7901 and BGC-823 cells were treated with the agent at 0,1,2,4,8,16 mg/mL for 24/48/72 h, and at the core experimental concentrations of 2,4,8 mg/mL for 48 h; the xenograft model mice were treated with the agent at 15 g/kg via gavage (0.2 mL/10g) once daily for 14 consecutive days, and the 5-Fu positive control group was treated with 5-Fu at 25 mg/kg via intraperitoneal injection every other day for 14 consecutive days	Associated with reduced PKM2 expression and nuclear translocation, lower aerobic glycolysis, and modulation of PKM2/HIF-1alpha-related markers	Sun et al. (2021)
Jian-pi-yang-zheng	Huangqi (Milkvetch Root, Astragalus mongholicus Bunge), Dangshen (Codonopsis Root, Codonopsis pilosula (Franch.) Nannf.), Sanleng (Common Burreed Tuber, Sparganium stoloniferum (Buch.-Ham. ex D.Don) Buch.-Ham.), Ezhu (Zedoary Rhizome, Curcuma phaeocaulis Val.)	*In vitro*: SGC-7901, MGC-803, BGC-823 cells and human monocytic leukemia cells (THP-1); *in vivo*: BALB/c nude mice xenografted with human gastric cancer cells (SGC-7901, MGC-803) and patient-derived xenograft mice Dose/concentration range: AGS and MKN28 cells were treated with the agent at 50 nM for 24 h; THP-1 cells in co-culture were treated with exosomes at 10 mg/mL for 48 h; the xenograft model mice were given NC exo/KD exo at 10 mg per mouse via intratumoral injection every other day until the experimental endpoint, and the GdCl3 group was treated with GdCl3 at 30 mg/kg via intravenous injection every 4 days in synchronization with exosome intervention	Associated with reduced M2-like tumor-associated macrophage infiltration and modulation of PKM2/HIF-1α-related markers	Yuan (2024)
Jianpi Huyu Jiedu Formula	Huangqi (Milkvetch Root, Astragalus membranaceus (Fisch.) Bunge), Taizishen (Heterophylly Falsestarwort Root, Pseudostellaria heterophylla (Miq.) Pax ex Pax et Hoffm.), Baizhu (Atractylodes Rhizome, Atractylodes macrocephala Koidz.), Fuling (Poria Cocos, Poria cocos (Schw.) Wolf), Sanqi (Notoginseng Root, Panax notoginseng (Burkill) F.H.Chen ex C.How), Ezhu (Zedoary Rhizome, Curcuma phaeocaulis Val.), Baihua Sheshecao (Hedyotis Herb, Hedyotis diffusa Willd.), Shougong (Gecko, Gekko swinhonis Guenther), Houtougu (Lion’s Mane Mushroom, Hericium erinaceus (Bull.) Pers.)	*In vivo*: MNNG-induced GPL rat model and ATP4a−/− GPL mouse model Dose/concentration range: SD rats with GPL induced by MNNG + hunger-satiety disorder were treated with Jianpi Huayu Jiedu Formula at 4.5 g/kg/d, 9 g/kg/d and vitam at 270 mg/kg/d via gavage (once daily, 6 consecutive days a week, rest for 1 day) for 16 consecutive days starting from week 13; ATP4a−/− mice with GPL were treated with JPHYJDF at 7.5 g/kg/d, 15 g/kg/d and vitam at 0.2 g/kg/d via gavage (once daily, 6 consecutive days a week, rest for 1 day) for 10 consecutive days starting from week 11	Associated with changes in AKT/FOXO3a- and PI3K/AKT/HIF-1α-related markers, together with improvement of glycolysis/OXPHOS imbalance in GPL models	He (2022)
Wei piling	Huangqi (Milkvetch Root, Astragalus membranaceus (Fisch.) Bunge), Taizishen (Heterophylly Falsestarwort Root, Pseudostellaria heterophylla (Miq.) Pax), Baizhu (Atractylodes Rhizome, Atractylodes macrocephala Koidz.), Fuling (Poria Cocos, Poria cocos (Schw.) Wolf), Sanqi (Notoginseng Root, Panax notoginseng (Burkill) F.H.Chen ex C.How), Ezhu (Zedoary Rhizome, Curcuma phaeocaulis Val.), Sheshecao (Hedyotis Herb, Hedyotis diffusa Willd.), Hougujun (Lion’s Mane Mushroom, Hericium erinaceus (Bull.) Pers.), Shougong (Gecko, Gekko swinhonis Guenther)	*In vivo*: ATP4a−/− mouse model Dose/concentration range: Atp4a−/− mice (GPL model) were treated with Wei piling at 0, 7.5 g/kg/d, 15 g/kg/d and Vitamin U at 0.2 g/kg/d via intragastric gavage (once daily) for 10 consecutive weeks starting from week 10 of age	Reported to delay GPL progression in association with modulation of mTOR/HIF-1α/SIRT6-related markers	Liu (2019)
Xiaojianzhong Decoction	Guizhi (Cinnamon Twig, Cinnamomum cassia (L.) J.Presl), Gancao (Licorice Root, Glycyrrhiza uralensis Fisch.), Dazao (Chinese Jujube Fruit, Ziziphus jujuba Mill.), Shaoyao (Peony Root, Paeonia lactiflora Pall.), Shengjiang (Fresh Ginger Rhizome, Zingiber officinale Roscoe), Jiaoyi (Maltose, maltose)	*In vivo*: MNNG-induced GPL rat model Dose/concentration range: Wistar rats (GPL model induced by MNNG + hunger-satiety alternation) were treated with Xiaojianzhong Decoction at 0, 1.875 g/kg/d, 3.75 g/kg/d, 7.50 g/kg/d and Tretinoin at 0.04 g/kg/d via intragastric gavage (once daily) for 4 consecutive weeks after 18 weeks of modeling	Associated with reduced autophagy- and glycolysis-related markers, improved mucosal hypoxia, and modulation of PI3K/AKT/mTOR- and p53/AMPK/ULK1-related proteins in an MNNG-induced GPL rat model	Zhang F. et al. (2023)
Modified Jian-pi-yang-zheng	Huangqi (Astragali Radix, Astragalus membranaceus (Fisch.) Bunge [Leguminosae]), Dangshen (Codonopsis Radix, Codonopsis pilosula (Franch.) Nannf. [Campanulaceae]), Sanleng (Sparganii Rhizoma, Sparganium stoloniferum (Buch.-Ham. ex Graebn.) Buch.-Ham. [Sparganiaceae]), Ezhu (Curcumae Rhizoma, Curcuma zedoaria (Christm.) Roscoe [Zingiberaceae])	*In vitro*: MGC-803, SGC-7901, BGC-823; *in vivo*: xenograft tumor mouse model (MFC murine gastric cancer cells, SGC-7901 human gastric cancer cells) Dose/concentration range: MGC-803, SGC-7901, BGC-823 cells were treated with modified Jianpi Yangzheng Decoction at 0, 2, 4, 8 mg/mL for 48 h	Associated with apoptosis of gastric cancer cells, reduced PKM2-related signaling, and modulation of PI3K/Akt/mTOR-related markers	Wu J. et al. (2022)
Jianpi Yangzheng Xiaozheng Formula	Dangshen (Codonopsis Root, Codonopsis pilosula (Franch.) Nannf.), Shenghuangqi (Milkvetch Root, Astragalus membranaceus (Fisch.) Bunge), Chaobaizhu (Bran-fried Atractylodes Rhizome, Atractylodes macrocephala Koidz.), Chaobaishao (Stir-fried White Peony Root, Paeonia lactiflora Pall.), Danggui (Chinese Angelica Root, Angelica sinensis (Oliv.) Diels), Chenpi (Dried Tangerine Peel, Citrus reticulata Blanco), Ezhu (Zedoary Rhizome, Curcuma phaeocaulis Val.), Sanleng (Common Burreed Tuber, Sparganium stoloniferum (Buch.-Ham. ex D.Don) Buch.-Ham.), Shijianchuan (Chinese Sage Herb, Salvia chinensis Benth.), Baihua Sheshecao (Hedyotis Herb, Hedyotis diffusa Willd.), Shenggancao (Licorice Root, Glycyrrhiza uralensis Fisch.)	*In vitro*: HGC-27 cells; *in vivo*: BALB/c nude mouse model with HGC-27 xenograft tumors Dose/concentration range: Human gastric cancer HGC-27 cells were treated with Jianpi Yangzheng Xiaozheng Formula at 0, 2, 4, 8 mg/mL for 24 h; BALB/c nude mice bearing HGC-27 xenografts were treated with Jianpi Yangzheng Xiaozheng Formula at 20 g/kg via intragastric gavage (once daily) for 14 consecutive days	Associated with reduced glycolysis-related readouts and attenuation of malignant progression in GC models	Tao (2020)
Compound Lizard Powder	Midian Maxi (*Eremias multiocellata*, *Eremias multiocellata* Günther), Huangqi (Milkvetch Root, Astragalus membranaceus (Fisch.) Bunge), Baizhu (Atractylodes Rhizome, Atractylodes macrocephala Koidz.), Banzhilian (Scutellariae Barbatae Herba, Scutellaria barbata D.Don), Yanhusuo (Corydalis Rhizome, Corydalis yanhusuo W.T.Wang ex Z.Y.Su and C.Y.Wu), Shanzha (Hawthorn Fruit, Crataegus pinnatifida Bunge), Baishao (White Peony Root, Paeonia lactiflora Pall.), Shanyao (Chinese Yam Rhizome, Dioscorea polystachya Turcz.), Lujiaojiao (Deer Horn Glue, Cervus nippon Temminck/*Cervus elaphus* Linnaeus), Wumei (Dark Plum Fruit, Prunus mume (Siebold and Zucc.) Siebold and Zucc.), Gancao (Licorice Root, Glycyrrhiza uralensis Fisch.)	*In vivo*: MKN45/DDP cell-induced cisplatin-resistant gastric cancer nude mouse xenograft model Dose/concentration range: BALB/c nude mice were randomly divided into model group, cisplatin group (0.002 g/kg, intraperitoneal injection twice a week), Compound lizard powder group (2.8 g/kg, intragastric gavage twice a day), and combination group (the same doses as the single drug groups). All groups were treated for 4 consecutive weeks; the model group was given an equal volume of normal saline by gavage synchronously	Associated with reduced PI3K/Akt activation markers, lower expression of glycolysis-related proteins (GLUT1, LDHA, HK2, PKM2) and multidrug resistance-related proteins/genes (MRP1, P-gp), and reduced lactate production in cisplatin-resistant GC models	Cheng et al. (2024)
Weipixiao decoction	Huangqi (Astragali Radix, Astragalus membranaceus (Fisch.) Bunge), Taizishen (Radix Pseudostellariae, Pseudostellaria heterophylla (Miq.) Pax), Baizhu (Atractylodis Macrocephalae Rhizoma, Atractylodes macrocephala Koidz.), Danshen (Salviae Miltiorrhizae Radix et Rhizoma, Salvia miltiorrhiza Bunge), Baihua Sheshecao (Hedyotis Diffusae Herba, Hedyotis diffusa (Willd.) Roxb.)	*In vivo*: MNNG-induced GPL rats Dose/concentration range: SD rats were randomly divided into normal group, model group, positive control group, low-dose Weipixiao decoction group (9.9 g/kg/d), and high-dose Weipixiao decoction group (19.8 g/kg/d). The model group was induced with gastric precancerous lesions by *ad libitum* drinking of 200 mg/L MNNG solution and alternate-day feeding for 16 weeks. After successful modeling, each drug group was treated via intragastric gavage once daily for 10 consecutive weeks; the normal group and model group were given equal volume of distilled water synchronously	Associated with upregulation of miR-34a and modulation of PI3K/AKT/mTOR-related markers in MNNG-induced GPL rats	Cai et al. (2019)
Zuojin Pill	Huanglian (Coptidis Rhizoma, Coptis chinensis Franch.), Wuzhuyu (Evodiae Fructus, Evodia rutaecarpa (Juss.) Benth.)	*In vitro*: Human gastric cancer cisplatin-resistant cell line SGC-7901/DDP Dose/concentration range: SGC-7901/DDP cells (cisplatin-resistant gastric cancer cells) were treated with Zuojin Pill at 25,50,100 μg/ml for 12,24,48 h	Associated with downregulation of c-Myc, HIF-1α, GLUT1, LDHA, and HK2 in cisplatin-resistant GC cells	Sun et al. (2019a)
Modified Zuojin Pill	Huangqi (Astragali Radix, Astragalus mongholicus Bunge), Chenpi (Citri Reticulatae Pericarpium, Citrus reticulata Blanco), Sanqi (Notoginseng Radix et Rhizoma, Panax notoginseng (Burkill) F.H.Chen), Huanglian (Coptidis Rhizoma, Coptis chinensis Franch.), Wuzhuyu (Evodiae Fructus, Tetradium ruticarpum (A.Juss.) T.G.Hartley), Gancao (Glycyrrhizae Radix et Rhizoma, Glycyrrhiza glabra L.)	*In vitro*: GES-1 cells; *in vivo*: MNNG-induced GPL rats Dose/concentration range: GES-1 cells were treated with 10% serum containing Modified Zuojin Pill at low (0.63 g/kg corresponding serum), medium (1.26 g/kg corresponding serum), high (2.51 g/kg corresponding serum) doses for 48h	Associated with downregulation of HIF-1α, GLUT1, HK2, PFKFB3, PKM2, and LDHA in GPL models	Liu Z. et al. (2025)
Zuojin Pill	Huanglian (Coptidis Rhizoma, Coptis chinensis Franch.), Wuzhuyu (Evodiae Fructus, Evodia rutaecarpa (Juss.) Benth.)	*In vitro*: SGC-7901 cells Dose/concentration range: SGC-7901 cells were treated with alcohol extract of Zuojin Pills at 5,10,25,50,100,200 μg/mL for 24/48/72 h	Associated with reduced glucose uptake, lactate production, and ATP content, and downregulation of RTKN in GC cells	Wu et al. (2021)

## 4 Modulation of glycolytic enzymes in gastric precancerous lesions and gastric cancer

### 4.1 HK2

Aberrant glucose metabolism, particularly aerobic-condition glycolysis, is a hallmark of GC progression. The expression of HK2 is significantly increased in various malignant tumors and is closely associated with the enhancement of aerobic-condition glycolysis. Notably, overexpression of HK2 was a common phenomenon in GC cases with poor prognosis (Rho et al., 2007). Compared with other subtypes, HK2 exhibited higher efficiency in promoting aerobic-condition glycolysis (Ciscato et al., 2021). Emerging studies have revealed that various plant metabolites and TCM formulas inhibit glycolysis in GC by modulating HK2 and its regulatory pathways, offering novel directions for metabolic intervention in this disease. Many plant metabolites, such as DT-13, exert anti-tumor effects through modulating HK2. DT-13, a saponin metabolite isolated from Sanqi (Notoginseng Root, *Panax notoginseng* (Burkill) F.H.Chen ex C.How, exerted a synergistic effect with topotecan by promoting epidermal growth factor receptor (EGFR) degradation via non-myosin IIA activity. This dual mechanism not only directly inhibited the enzyme activity of HK2, but also disrupted the specific binding of HK2 to mitochondria, thereby blocking the energy transfer of glycolytic intermediates to mitochondria and weakening the efficiency of aerobic-condition glycolysis. Consequently, this intervention depleted energy supply and inhibited the malignant proliferation of GC cells (Yu et al., 2019). Emodin, a major active anthraquinone metabolite extracted from Dahuang (Chinese rhubarb, *Rheum palmatum L*.), has anti-inflammatory effects (Liu Y. et al., 2022). Emodin demonstrated concentration-dependent inhibitory effects on MGC803 and BGC-823 GC cells. It downregulated HK2 protein expression, reduced glucose consumption and lactate production, and upregulated the pro-apoptotic protein Bax to induce cell apoptosis. Notably, glycolytic inhibition triggered a cascade downregulation of the oncogene FOXD1, forming a “metabolism-transcription” double inhibition network that synergistically suppressed the proliferation, migration, and invasion of GC cells (Liu C. H. et al., 2025; Guan et al., 2018). Luteolin, a natural flavonoid widely distributed in various plants such as celery, broccoli, and *Chrysanthemum × morifolium* (Ramat.) Hemsl., induced apoptosis in AGS GC cells by coordinately downregulating key glycolytic molecules such as HK2, GLUT1, and PKM2, blocking glycolysis at multiple nodes to inhibit glucose uptake and lactate production (Shang et al., 2023). Rosmarinic acid suppressed the Warburg effect and tumor xenograft growth by reducing interleukin-6 (IL-6) levels in the tumor microenvironment, inhibiting STAT-3α activation, and downregulating HK2 and LDHA expression (Wei et al., 2011). Additionally, matrine inhibited the metastasis and invasion of HGC-27 cells by improving their metabolic phenotype, simultaneously suppressing the expression of HK and the tricarboxylic acid cycle, which disrupts both glycolytic energy supply and mitochondrial metabolism (Zeng and Lu, 2021).

Traditional Chinese medicine (TCM) formulas consist of botanical drugs, animal ingredients, and mineral drugs. They have shown advantages in regulating the HK2 pathway, with typical representatives including Jianpi Yangzheng Xiaozheng Decoction and Xiaotan Sanjie Formula. Jianpi Yangzheng Xiaozheng Decoction comprises *Codonopsis pilosula* (Franch.) Nannf., *Astragalus membranaceus* (Fisch.) Bunge, *Atractylodes macrocephala* Koidz., *Paeonia lactiflora* Pall., *Angelica sinensis* (Oliv.) Diels, *Salvia chinensis* Benth., *Sparganium stoloniferum* (Buch.-Ham. ex D.Don) Buch.-Ham., *Curcuma phaeocaulis* Val., *Hedyotis diffusa* Willd., *Citrus reticulata* Blanco and *Glycyrrhiza uralensis* Fisch. As a TCM Formula, it was reported in GC models to be associated with reduced HK2 expression, decreased glucose consumption and lactate production, and increased apoptosis. These effects were accompanied by changes in PI3K/AKT/mTOR/HIF-1α/c-Myc-related signaling markers, but direct pathway targeting has not been conclusively established (Yang, 2016). Xiaotan Sanjie Formula consists of *Arisaema erubescens* (Wall.) Schott, *Pinellia ternata* (Thunb.) Breit., *Buthus martensii* Karsch, *Scolopendra subspinipes* mutilans L. Koch, *Poria cocos* (Schw.) Wolf and *G. uralensis* Fisch. This formula, evaluated in an MKN-45 xenograft model, was associated with reduced HK2 activity/expression in serum and tumor tissues, lower glycolysis-related readouts, and suppressed tumor growth. These findings suggested an HK2-associated metabolic effect rather than definitive evidence of direct energy-deprivation targeting (Ruan et al., 2021).

Taken together, HK2 acts as a core regulatory node in aerobic-condition glycolysis of GC cells, and its abnormal overexpression is tightly linked to the malignant progression and poor prognosis of GC. Plant metabolites and TCM formulas with multi-metabolite and multi-target characteristics have showed prominent advantages in modulating glycolysis through HK2-associated mechanisms, providing valuable candidate strategies and novel research ideas for the development of metabolic intervention therapies for GPL and GC. Across the HK2-related studies summarized in Table 1, most claims are supported by reduced glucose consumption/lactate release and downregulated HK2 expression after exposure to a plant metabolite or multi-botanical preparation. However, HK2 expression changes alone do not establish HK2 as the causal pharmacological target. Key limitations include: (i) infrequent use of direct HK activity assays, rescue experiments (HK2 overexpression), or mitochondria-binding readouts to demonstrate specific HK2 engagement; (ii) limited reporting of concentration–response relationships and whether tested concentrations are pharmacologically plausible; (iii) incomplete reporting of vehicle/positive controls and exposure durations; and (iv) for plant metabolites and botanical drug, frequent absence of voucher specimens and quantitative chemical profiles, limiting reproducibility. Therefore, the current evidence can support “HK2-associated glycolysis modulation in GC models” but cannot, in most cases, support “HK2-modulated therapy” claims or clinical translation.

### 4.2 PKM2

Another rate-limiting enzyme in glycolysis is PK, which exists in four subtypes, namely PKL, PKR, PKM1, and PKM2 (Dong et al., 2016). The absence of PKM2 in GC cells led to a significant reduction in their proliferative and invasive phenotypes (Kitayama et al., 2017). Notably, PKM2 plays a crucial role in the initiation and progression of GC, thereby emerging as a core target for plant metabolites and TCM formulas intervention research. The plant metabolite salidroside exerted a dose-dependent inhibitory effect on the proliferation of human GC cells. It promoted GC cell apoptosis and blocked the glycolytic pathway by downregulating the expression levels of PKM2, enolase 1 (ENO1), and glucose transporter 1 (GLUT1) (Dai et al., 2021).

Modified Jian-pi-yang-zheng (mJPYZ) consists of *C. pilosula* (Franch.) Nannf., *Astragalus mongholicus* Bunge, *S. stoloniferum* (Buch.-Ham. ex D.Don) Buch.-Ham., and *C. phaeocaulis* Val. Studies have confirmed that mJPYZ is reported to reduce PKM2 and HIF1α expression, lower glycolytic activity, and inhibit EMT-related phenotypes in GC models. The available evidence supports PKM2/HIF1α-associated metabolic modulation, although direct target engagement remains to be validated (Sun et al., 2021). Furthermore, this formula exerted pleiotropic effects beyond direct action on tumor cells. It also reduced the transfer of PKM2 from tumor cell-derived exosomes to macrophages, reshaped the energy metabolism profile of macrophages, and inhibited their polarization toward the pro-tumorigenic M2 phenotype. Collectively, these effects further curbed cancer progression at the level of tumor microenvironment regulation (Yuan, 2024).

Taken together, PKM2, a key rate-limiting enzyme in GC glycolysis, is closely associated with GC initiation and progression via its aberrant expression, and is investigated as a potential target for plant metabolites and TCM formulas to intervene in GC metabolism and suppress tumor progression. Although PKM2 is commonly recognized as a glycolytic node in GC, many included studies infer PKM2 modulation based on changes in PKM2 protein/mRNA levels or downstream markers, rather than validated PKM2 activity states (tetramer vs. dimer), phosphorylation status, or flux-based measurements. Studies relying on single cell lines, single concentrations, or short exposure times fail to distinguish pathway-specific modulation from generalized growth inhibition. Stronger evidence would require three key elements: (i) concentration–response data accompanied by cell viability controls, (ii) direct enzymatic assays or structural/biophysical assessments of target engagement, and (iii) functional validation (e.g., genetic or pharmacological perturbation of PKM2) to confirm causality. In the absence of these, the data are best interpreted as “PKM2-correlated metabolic effects” rather than definitive evidence of PKM2 modulating.

### 4.3 LDH

LDH catalyzes the final step of glycolysis and mediates the interconversion between lactate and pyruvate (Sharma et al., 2022). It comprises three isoforms, LDHA, LDHB, and LDHC, each executing distinct biological functions. Specifically, LDHA drives the conversion of pyruvate to lactate (Feng et al., 2018), whereas LDHB promotes the reverse reaction (Urbańska and Orzechowski, 2019). Accumulating evidence has demonstrated that LDH and its isoenzymes play a crucial role in metabolic reprogramming during the development and progression of GC (Liu et al., 2015). Kirenol is a bioactive metabolite isolated from Xixian (Oriental siegesbeckia, Sigesbeckia orientalis L.) (Xiao et al., 2023). Research has shown that kirenol can reduce oxidative stress and inflammation levels through the NF-κB signaling pathway. It significantly inhibited LDH expression, thereby reducing tumor volume and incidence, without causing adverse effects on body weight (Liu et al., 2020a). Catechins, the major bioactive plant metabolites in green tea, exhibit prominent anti-cancer and anti-angiogenic activities (Mizushina et al., 2011). Specifically, catechins bound to key amino acid residues such as T94 and A95 in LDHA, which interfered with the interaction between LDHA and its pyruvate substrate. This binding event effectively decreased LDHA activity and lactate production in SNU620/5-FU cells, ultimately reversing the drug resistance of tumor cells to 5-FU (Han et al., 2021). Tanshinone IIA, a pharmacologically active metabolite derived from the rhizome of the Danshen (Salviae Miltiorrhizae Radix et Rhizoma, *Salvia miltiorrhiza* Bunge), has been documented to exhibit anti-cancer activity (Guan et al., 2020). Under anaerobic microenvironments, tanshinone IIA downregulated the expression of key glycolytic enzymes, including LDHA and glyceraldehyde-3-phosphate dehydrogenase (GAPDH). This downregulation further inhibited glucose uptake, lactate production, and adenosine triphosphate (ATP) synthesis, thereby suppressing the proliferation of MGC803 cells (Liu Z. et al., 2025). Taraxasterol, a pentacyclic triterpenoid plant sterol isolated from Pugongying (Mongolian dandelion, *Taraxacum mongolicum* Hand.-Mazz.), possesses diverse biological activities, including anti-tumor effects (Han et al., 2025). Mechanistically, taraxasterol modulated the glycerol-3-phosphate dehydrogenase 2 (GPD2)-mediated glycolytic pathway. By reducing LDH activity and inhibiting glucose metabolism as well as ATP production, taraxasterol effectively suppressed GC cell proliferation and induced cell apoptosis (Zhao et al., 2021).

Collectively, these studies have highlighted the pivotal regulatory functions of LDH and related glycolytic pathways in the pathogenesis of GC and have suggested the therapeutic potential of plant metabolites in modulating LDH-associated metabolic reprogramming. These findings provide an important theoretical basis and drug screening strategy for the development of highly effective and low-toxicity therapeutic approaches for GC. Given that LDH isoenzymes make distinct contributions to lactate–pyruvate interconversion, claims related to LDH require clear specification of the isoform examined and direct measurement of enzymatic activity. In the reviewed literature, LDHA is frequently implicated. However, many studies do not directly measure LDHA activity or distinguish between effects on enzyme activity versus expression. A more robust mechanistic framework emerges when studies integrate enzyme kinetics or binding assays, cellular metabolic profiles, and genetic validation. Nonetheless, achievable drug exposure levels and *in vivo* relevance still require further clarification. Overall, LDH represents a plausible therapeutic target, but the current evidence base remains inconsistent and often underpowered to support definitive mechanistic conclusions.

### 4.4 GLUT1

GLUT-1, the first identified member of the glucose transporter superfamily, belongs to the solute carrier 2A (SLC2A) family. It mediates energy-independent transport of glucose across hydrophobic cell membranes along concentration gradients (Macheda et al., 2005). Inhibition of GLUT-1 expression reversed the Warburg effect in MKN45 cells and induced apoptosis (Zhang et al., 2015). As a key regulator of glycolytic metabolism in GC cells, GLUT-1 has emerged as a critical therapeutic target for pharmacological intervention. *In vitro* studies have revealed a unique mechanism by which cardiac glycosides act on MKN45 cells. These metabolites reduced the cell-surface abundance of GLUT-1 by inducing dynamin-dependent endocytosis, thereby modulating GLUT-1 for lysosomal degradation. This regulatory pattern significantly impaired glucose uptake by cancer cells and effectively inhibited glycolysis in GC cells (Fujii et al., 2022). Celastrol, a bioactive molecule extracted from the plant Leigongteng (Thunder God Vine, *Tripterygium wilfordii* Hook.f.), possesses anti-inflammatory and anti-cancer properties (Wang D. et al., 2024). Celastrol employed a multi-modulate synergistic approach. It not only modulated GLUT1 but also suppressed the expression of HK2 and LDH, blocking aerobic-condition glycolysis in BGC-823 cells. This mechanism cut off the energy supply and raw materials for biomacromolecule synthesis in GC cells, interfering with the normal progression of the cell cycle, inducing apoptosis, and effectively inhibiting the proliferation of GC cells (Li K. et al., 2019). These findings not only revealed the crucial role of GLUT1 in the metabolic regulation of GC but also provided important theoretical support and directions for developing new therapeutic strategies modulating GLUT1 in GC treatment.

Although GLUT1 is frequently employed as a marker of elevated glucose uptake, alterations in its expression only serve as an indirect indicator. To make robust conclusions that a metabolite inhibits glucose transport, studies should incorporate functional glucose uptake assays, rigorous normalization to cell number or viability, and ideally, genetic or pharmacological specificity controls. Since many of the included studies lack these critical data, GLUT1-related observations should generally be interpreted as supportive correlations rather than definitive mechanistic endpoints.

## 5 Modulation of multiple signaling pathway in gastric precancerous lesions and gastric cancer

### 5.1 PI3K/AKT/mTOR

The PI3K/AKT/mammalian target of rapamycin (mTOR) signaling pathway is intricately involved in the regulation of cell growth and proliferation (Khezri et al., 2022). PI3Ks are plasma membrane-associated lipid kinases composed of two regulatory subunits (p85 and p55) and one catalytic subunit (p110) (Yang et al., 2019). Based on their structural features and substrate specificities, the PI3K family can be categorized into three major classes: Class I, Class II, and Class III. Notably, Class I PI3K is the primary subtype implicated in oncogenic signaling and serves as the main mediator responsible for the activation of the downstream serine/threonine kinase AKT (Nicholson and Anderson, 2002). In contrast, Class II and Class III PI3Ks are mainly implicated in intracellular membrane trafficking and autophagy, with relatively limited direct roles in the malignant progression of cancer (Soler et al., 2015; Morgos et al., 2024). AKT is involved in a variety of cellular processes, including glucose metabolism, cell proliferation, and cell survival. As a downstream kinase of the PI3K/AKT pathway, mTOR forms two types of multiprotein complexes: mTOR complex 1 (mTORC1) and mTOR complex 2 (mTORC2) (Deng et al., 2023). The activation of mTORC1 regulates cell growth by modulating protein synthesis, ribosome biogenesis, and autophagy (Wullschleger et al., 2006). The mTORC2 pathway, together with PDK1 and PI3K, plays a critical role in the activation of AKT. Notably, PI3K represents a potential drug target in cancers with dysregulated AKT signaling (Kim et al., 2017). In most GPL and GC studies, including the evidence summarized below, the regulatory effects of plant metabolites and TCM formulas on the PI3K/AKT/mTOR pathway are primarily directed at the mTORC1 branch, which serves as the major mediator of the pathway’s metabolic and proliferative functions during the malignant transformation of the gastric mucosa. Collectively, the PI3K/AKT/mTOR signaling pathway plays a key role in the reprogramming of glucose metabolism in GPL and GC.

Jianpi Huyu Jiedu Formula comprises *A. membranaceus* (Fisch.) Bunge, *Pseudostellaria heterophylla* (Miq.) Pax ex Pax et Hoffm., *A. macrocephala* Koidz., *P. cocos* (Schw.) Wolf, *P. notoginseng* (Burkill) F.H.Chen ex C.How, *C. phaeocaulis* Val., *H. diffusa* Willd., *Gekko swinhonis* Guenther, and *Hericium erinaceus* (Bull.) Pers. In GPL, studies have shown that Jianpi Huyu Jiedu Formula was associated with changes in AKT/FOXO3a- and PI3K/AKT/HIF-1α-related protein markers, together with reduced LDHA expression and improvement of hypoxia- and oxidative stress-related readouts. These data suggest pathway-associated metabolic modulation rather than definitive proof of direct pathway inhibition (He, 2022). On the other hand, it is accompanied by the downregulation of the PI3K/AKT/HIF-1α pathway and a subsequent reduction in the expression of the glycolytic enzyme LDHA (Pan et al., 2020). Wei piling consists of *A. membranaceus* (Fisch.) Bunge, *P. heterophylla* (Miq.) Pax, *A. macrocephala* Koidz., *P. cocos* (Schw.) Wolf, *P. notoginseng* (Burkill) F.H.Chen ex C.How, *C. phaeocaulis* Val., *H. diffusa* Willd., *H. erinaceus* (Bull.) Pers., and *G. swinhonis* Guenther. This formula was reported in GPL models to ameliorate lesion progression in association with changes in mTOR/HIF-1α/SIRT6-related markers (Liu, 2019). Xiaojianzhong Decoction contains *Cinnamomum cassia* (L.) J.Presl, *G. uralensis* Fisch., *Ziziphus jujuba* Mill., *P. lactiflora* Pall., *Zingiber officinale* Roscoe, and maltose. It was associated with improvement of gastric mucosal pathology, reduced autophagy- and glycolysis-related readouts, and modulation of PI3K/AKT/mTOR- and p53/AMPK/ULK1-related proteins. Because this evidence derives from a single animal study, direct pathway targeting cannot be inferred (Zhang J. X. et al., 2023).

In GC, the PI3K/AKT/mTOR pathway is involved in glycolysis, microenvironmental regulation, and drug resistance (Xing et al., 2024; Zhang et al., 2021). A variety of plant metabolites exert regulatory effects on GC progression. Licorice is the root of *G. uralensis* Fisch. and possesses anti-inflammatory, anti-tumor, and antibacterial properties (Dang et al., 2024). Liquiritigenin A, a natural flavonoid metabolite, is derived from *G. uralensis* Fisch. root (Liu et al., 2013; Jiang et al., 2020). Studies have shown that plant metabolites such as licoflavone A are associated with the inhibition of HK2-mediated glycolysis and the induction of GC cell apoptosis, with concomitant downregulation of the AKT signaling cascade (Wu et al., 2018). Juglone blocked the GC cell cycle and reduced glycolytic activity, which was accompanied by the inhibition of the AKT/mTOR pathway (Yu and Jin, 2022). Arbutin reduced glucose transport and the expression of key glycolytic enzymes in GC cells in a PI3K/AKT/GLUT1 axis-associated manner (Han et al., 2024).

Numerous TCM formulas also modulate GC progression. mJPYZ was also associated with reduced exosomal transfer of PKM2 from tumor cells to macrophages and attenuation of M2-like polarization, together with changes in PI3K/AKT/mTOR-related markers (Wu J. et al., 2022). Jianpi Yangzheng Xiaozheng Formula comprises Codonopsis pilosula (Franch.) Nannf., Astragalus membranaceus (Fisch.) Bunge, Atractylodes macrocephala Koidz., Paeonia lactiflora Pall., Angelica sinensis (Oliv.) Diels, Citrus reticulata Blanco, Curcuma phaeocaulis Val., Sparganium stoloniferum (Buch.-Ham. ex D.Don) Buch.-Ham., Salvia chinensis Benth., Hedyotis diffusa Willd., and Glycyrrhiza uralensis Fisch. It induced GC cell apoptosis and chemosensitivity, possibly by downregulating aerobic-condition glycolysis and stemness. This formula was accompanied by the inhibition of the PI3K/AKT/mTOR/HIF1-α/c-Myc cascade, a subsequent reduction in glycolytic enzyme expression, suppressed glycolysis, and diminished cell stemness (Tao, 2020). Compound Lizard Powder contains *Eremias multiocellata Günther*, *A. membranaceus* (Fisch.) Bunge, *A. macrocephala* Koidz., *Scutellaria barbata* D.Don, *Corydalis yanhusuo* W.T.Wang ex Z.Y.Su and C.Y.Wu, *Crataegus pinnatifida* Bunge, *P. lactiflora* Pall., *Dioscorea polystachya* Turcz., *Cervus nippon* Temminck/*Cervus elaphus* Linnaeus, *Prunus mume* (Siebold and Zucc.) Siebold and Zucc., and *G. uralensis* Fisch. It was reported in cisplatin-resistant GC models to reduce glycolysis-related proteins and PI3K/Akt activation markers, in association with decreased resistance-related phenotypes. These data do not yet establish direct inhibition of the PI3K/Akt pathway (Cheng et al., 2024).

In summary, studies summarized in Tables 1, 2 report that plant metabolites and TCM formulas are associated with reduced phosphorylation levels of the PI3K/AKT/mTOR signaling cascade in GPL and GC models, accompanied by decreased glycolytic activity. However, these findings are correlative rather than causal in the majority of studies, and definitive claims of “targeting” the PI3K/AKT/mTOR pathway are not supported by current evidence. Specifically, most relevant studies only detect the expression of p-PI3K, p-AKT and p-mTOR via Western blot, without functional validation experiments, direct kinase activity detection, or glycolytic flux-based metabolic profiling. For example, the conclusion that arbutin inhibits glycolysis via the PI3K/AKT/GLUT1 axis is only based on the downregulation of pathway protein expression, with no verification of AKT kinase activity or glucose transport flux. Thus, the observed changes in PI3K/AKT/mTOR signaling are likely secondary to general cellular growth inhibition or cytotoxicity rather than specific target engagement by plant metabolites or TCM formulas. All above observations should therefore be strictly described as PI3K/AKT/mTOR-associated metabolic modulation rather than direct targeting.

### 5.2 Hippo

The Hippo pathway is highly conserved signaling throughout *Drosophila melanogaster* to mammals, serving as a master regulator of organ size and tissue regeneration (Harvey et al., 2003). Over the past decade, accumulating evidence has supported the involvement of Hippo pathway dysfunction, particularly the dysregulation of core Hippo signaling effectors YAP1 and TAZ, in tumor progression and recurrence (Liu X. et al., 2022). Studies have shown that the Hippo pathway is closely related to glucose homeostasis, and aerobic-condition glycolysis can enhance the transcriptional activity of YAP/TAZ (Enzo et al., 2015; Wang et al., 2015). Gypenoside, the main active metabolite of Jiaogulan (Gynostemma pentaphyllum, *Gynostemma pentaphyllum* (Thunb.) Makino), plays a vital role in regulating glycometabolism disorders (Xie et al., 2023). Gypenoside suppressed through the Hippo pathway, likely by inhibiting the phosphorylation and nuclear translocation of YAP, a downstream effector of Hippo signaling (Pan et al., 2024). By decreasing YAP protein levels, oleanolic acid inhibited the transcription of HIF-1α and its downstream glycolytic effectors, such as HK2 and PFK1, thereby reducing glucose consumption and lactate production (Li Y. et al., 2019).

Collectively, these studies have established the Hippo-YAP axis as a pivotal node integrating cell proliferation and glycolytic reprogramming in GC. Plant metabolites capable of interfering with this pathway exert dual effects, including inhibiting carcinogenic signal transduction and controlling abnormal energy metabolism. Future studies investigating the crosstalk between the Hippo pathway and other metabolic pathways, such as PI3K/AKT and AMPK, may uncover synergistic therapeutic strategies, particularly for patients with hyperactive YAP signaling or metabolism-driven tumors.

Modulation of the Hippo pathway by plant metabolites and TCM formulas has been reported in a limited subset of GC models, typically based on changes in YAP/TAZ signaling and associated alterations in glycolysis-related markers. Given that Hippo pathway readouts are sensitive to cell density, cytoskeletal stress, and general cellular perturbations, studies should control for these confounding factors and provide functional validation in addition to metabolic endpoints. Such validation is scarce in the current evidence base. Therefore, claims related to the Hippo pathway should generally be considered as low-to-moderate confidence unless supported by rigorous causal experiments.

### 5.3 Non-coding RNAs

ncRNA include small interfering RNAs (siRNAs), microRNAs (miRNAs), long ncRNAs (lncRNAs), and antisense RNAs (asRNAs) (Mirzaei and Hamblin, 2020). In recent years, ncRNAs have emerged as critical regulators in this process by modulating glycolytic metabolism (Zhu et al., 2020). The progression of GPL to malignant tumors is closely associated with metabolic reprogramming, particularly glycolytic pathway activation. Ginsenoside Rg3, one of the most potent plant metabolites extracted from the roots of the Renshen (Asian ginseng, *Panax ginseng* C.A.Mey.), has prominent roles in anti-tumor and anti-inflammation (Ren et al., 2021; Li S. L. et al., 2025). In GPL models, ginsenoside Rg3 inhibited glycolysis through the PI3K/AKT/miR-21 pathway, significantly suppressing cell proliferation and inducing apoptosis. This highlighted miRNA-21 as a key node for blocking precancerous progression (Liu et al., 2020b). Astragaloside IV, a major metabolite of Huangqi (Milkvetch Root, *A. membranaceus* (Fisch.) Bunge), has been demonstrated to exhibit preventive effects against various diseases, including cancers (Zhu and Lu, 2024). It exerted its effects via the miR-34a/LDHA axis, downregulating key glycolytic molecules such as LDHA and HIF-1α, while upregulating the tumor suppressor p53 to reverse gastric mucosal dysplasia and intestinal metaplasia (Zhang C. et al., 2018). In addition, the TCM formula Weipixiao Decoction, which consists of *A. membranaceus* (Fisch.) Bunge [Leguminosae], *P. heterophylla* (Miq.) Pax [Caryophyllaceae], *A. macrocephala* Koidz. [Asteraceae], *S. miltiorrhiza* Bunge [Lamiaceae], and *H. diffusa* (Willd.) Roxb. [Rubiaceae it was reported to exert protective effects in MNNG-induced GPL rats, including upregulation of miR-34a and modulation of PI3K/AKT/mTOR-related markers (Cai et al., 2019).

In GC, ncRNA-mediated regulatory networks are more complex. Rosmarinic acid, a natural polyphenolic metabolite, exhibited diverse pharmacological activities, notably its anti-cancer properties (Radziejewska et al., 2021). Rosmarinic acid inhibited the pro-inflammatory and oncogenic mediator miR-155, blocking the IL-6/STAT3 inflammatory pathway and consequently suppressing the Warburg effect in tumor cells (Han et al., 2015). As a tumor suppressor, miR-34a enhanced the sensitivity of GC cells to luteolin by directly modulating hexokinase HK1, providing a novel strategy for chemotherapy sensitization (Zhou et al., 2018). For clinical drug resistance, paeonol reversed apatinib resistance through the LINC00665/miR-665/MAPK1 axis. The long ncRNA LINC00665 acted as a molecular sponge for miR-665, relieving its inhibition on MAPK1, while paeonol disrupted this regulatory loop to restore apoptotic responses in resistant cells (Li et al., 2022). Schizandrin A, a natural metabolite derived from Wuweizi (Chinese magnoliavine, *Schisandra chinensis* (Turcz.) Baill., shows significant anticancer effects on multiple cancerous tissues (Pu et al., 2021). It inhibited GC cell viability and aerobic-condition glycolysis by upregulating miR-873–5p to suppress glucose-6-phosphate dehydrogenase (G6PD), blocking the pentose phosphate pathway and highlighting ncRNAs’ precision in metabolic regulation (Wang S. X. et al., 2024).

However, the aforementioned studies remain descriptive and preliminary, with limited rigorous evidence to confirm the specific, direct metabolic modulating of plant metabolites and TCM formulas via ncRNAs. Most studies lack comprehensive methodological validation, including clear target engagement, appropriate control groups, dose justification, and taxonomic and chemical authentication, all of which highlight substantial limitations that restrict translational confidence.

### 5.4 c-Myc

c-Myc, a pivotal oncogenic transcription factor, orchestrates metabolic reprogramming in cancer by integrating glycolysis and mitochondrial function (Yoshida, 2018; Grüning et al., 2010). Many studies have emphasized the various molecular mechanisms by which c-Myc regulates the progression of GC through energy metabolism pathways. Isoliquiritigenin, a flavonoid metabolite isolated from licorice root, shows important antitumor effects (Zhang X. R. et al., 2018). For instance, isoliquiritigenin suppressed GC growth by dual inhibition of GLUT4-mediated glucose uptake and disruption of the PDHK1/PGC-1α axis, with c-Myc and HIF-1α identified as downstream regulators of its anti-glycolytic effects. This metabolite reduced c-Myc protein expression, thereby inhibiting both aerobic-condition glycolysis and OXPHOS while inducing ROS accumulation, ultimately leading to tumor cell apoptosis (Yu et al., 2023). Podofilox, a medicinal metabolite extracted from various Berberidaceae plants, has been demonstrated by multiple experiments to possess antiviral and anticancer activities (Cohen et al., 2016). Podofilox exerted anti-proliferative effects by downregulating c-Myc alongside HK2, PKM2, and ATG10 in AGS and HGC-27 cells, linking c-Myc to the regulation of both glycolytic enzymes and autophagy-related genes in GC (An et al., 2021). β-asarone, the main active metabolite of the Chinese botanical drug *Acorus verus* (L.) Raf., exhibits a wide range of biological activities (Tao et al., 2020). β-asarone enhanced chemosensitivity to cisplatin by suppressing PDK1/PDK4-mediated glycolysis and reducing the expression of c-Myc and HIF-1α (Tao et al., 2020). β-asarone actd as a potent chemosensitiser for gastric cancer, while its carcinogenic potential and toxicological risks should be noted. Botanical drugs-derived active plant metabolites further illustrate c-Myc-centric metabolic regulation. Cardamonin from Hainanjiang (Hainan alpinia, *Alpinia hainanensis* K.Schum.) inhibited GC growth by modulating the c-Myc/GLUT4 axis, concurrently suppressing glycolysis and mitochondrial function to induce energy metabolic collapse (Liang, 2021). Shikonin is derived from Zicao (Gromwell, *Lithospermum eryothrorhizon*), can reduce cancer activity (Chang et al., 2022). Shikonin reduced lactate production and glycolytic flux by inhibiting the c-Myc/LDHA/HK2 pathway, thereby attenuating cell proliferation, migration, and invasion while promoting apoptosis (Zhang F. et al., 2023). As an isoquinoline alkaloid, berberine is distributed in plant families such as Berberidaceae, Ranunculaceae, and Papaveraceae, and is well-established for its diverse biological activities including anti-inflammatory and anticancer effects (Ayati et al., 2017). The combined use of berberine and Wnt/β-catenin inhibitors could synergistically reduce the expression of c-Myc, thereby inhibiting glycolysis and promoting cell apoptosis (Zhang et al., 2020). Notably, helichrysetin modulated the mTOR/p70S6K/c-Myc/PDHK1 axis to rewire energy metabolism, enhancing mitochondrial OXPHOS while suppressing glycolysis (Wang et al., 2022). Similarly, the TCM formula Zuojin Pill, composed of *Coptis chinensis* Franch. [Ranunculaceae] and *Evodia rutaecarpa* (Juss.) Benth. [Rutaceae], was reported to exert protective effects in MNNG-induced GPL rats, including upregulation of miR-34a and modulation of PI3K/AKT/mTOR-related markers (Sun et al., 2019a).

Overall, these findings indicate that c-Myc serves as the central hub of metabolic reprogramming in GC. Plant metabolites and TCM formulas exert therapeutic effects through multi-level regulation of c-Myc-related pathways. These mechanisms not only emphasize the complexity of GC metabolic adaptation, but also provide a strong theoretical basis for the development of c-Myc-modulated therapies that integrate metabolic inhibition with conventional chemotherapy or epigenetic regulation. Nevertheless, c-Myc is a broad transcriptional hub, and changes in its expression may be secondary to cellular stress responses. Reliable mechanistic conclusions require functional validation, such as c-Myc overexpression or knockdown rescue experiments, together with simultaneous metabolic flux measurements, which have not been uniformly and standardly applied in the existing literature.

### 5.5 HIF-1α

Due to the rapid proliferation and expansion of cancer cells, hypoxia exists in the core of tumor tissues (Feng et al., 2020). HIF-1α plays a key regulatory role in tumor cell proliferation, glucose metabolism, angiogenesis, invasion and metastasis, multidrug resistance, etc. (Méndez-Blanco et al., 2018). HIF-1α directly promotes the process of aerobic-condition glycolysis by enhancing the transcriptional expression of GLUT and other key glycolytic enzymes (Kim et al., 2006). HIF-1α serves as a pivotal regulator of glycolytic reprogramming in gastric carcinogenesis, with plant metabolite emerging as promising modulators of its signaling axis.

In GPL, celastrus orbiculatus extract further exemplified this regulatory mechanism by alleviating gastric mucosal lesions in GPL rats, decreasing lactate production, and suppressing the expression of HIF-1α, highlighting HIF-1α as a critical node in early-stage metabolic dysregulation (Zhang X. Z. et al., 2023). The TCM formula modified Zuojin Pill, consisting of *A. mongholicus* Bunge [Leguminosae], *C. reticulata* Blanco [Rutaceae], P*anax notoginseng* (Burkill) F.H.Chen [Araliaceae], *C. chinensis* Franch. [Ranunculaceae], *Tetradium ruticarpum* (A.Juss.) T.G.Hartley [Rutaceae], and *Glycyrrhiza glabra* L. [Fabaceae], inhibited glycolysis by suppressing the HIF-1α pathway, reducing glucose, lactate, and LDH levels in the gastric mucosa of GPL rats. Concurrently, it downregulated HIF-1α-associated proteins and mRNAs, reversing MNNG-induced malignant phenotypes in GES-1 cells (Liu S. et al., 2025).

The TCM formula modified Zuojin Pill inhibited glycolysis by suppressing the HIF-1α pathway, reducing glucose, lactate, and LDH levels in the gastric mucosa of GPL rats. Concurrently, it downregulated HIF-1α-associated proteins and mRNAs, reversing MNNG-induced malignant phenotypes in GES-1 cells (Liu S. et al., 2025).

In advanced GC, HIF-1α-driven glycolysis contributes to therapeutic resistance and tumor progression. Baicalein, another major metabolite of Huangqin (Baical skullcap, *Scutellaria baicalensis* Georgi), reversed hypoxia-induced 5-FU resistance in AGS cells by reducing HK2, LDH-A, PDK1 expression and disrupting the PTEN/AKT/HIF-1α signaling cascade. By promoting PTEN accumulation and suppressing AKT phosphorylation, baicalein diminished HIF-α expression under hypoxic conditions, restoring drug sensitivity (Chen et al., 2015). Liquiritigenin A exhibits anti-cancer effects under hypoxia by suppressing HIF-1α transcriptional activity (Dong et al., 2024). Similarly, aesculetin suppressed glucose uptake, lactate production, and HIF-1α expression in SGC-7901 cells, linking HIF-1α inhibition to reduced metastatic potential and glycolytic efficiency (Jia et al., 2024). Wogonin, a flavonoid derived from the botanical drug Huangqin (Baical skullcap, *S. baicalensis* Georgi), is renowned for its potent anti-cancer activity (Huang et al., 2012). It further reinforced this paradigm by downregulating HIF-1α and LDH, reducing lactate accumulation and alleviating the acidic tumor microenvironment (Wang et al., 2019).

However, the majority of the above studies remain descriptive and lack rigorous mechanistic validation. Most investigations fail to provide sufficient pharmacological details, standardized material characterization, or functional evidence to confirm specific HIF-1α modulating, which weakens the translational confidence of the current evidence.

### 5.6 ROS

Increased reactive oxygen species (ROS) production by tumorigenic cells leads to the upregulation of glycolysis, thereby contributing to the pathogenesis of diseases including cancer (Lebelo et al., 2019). ROS act as double-edged regulators in GC, balancing pro-oxidative stress and pro-survival signaling. Ginsenoside Rg3 exerted therapeutic effects in GPL by orchestrating ROS-mediated apoptosis and growth inhibition. In GPL rats, Rg3 alleviated intestinal metaplasia and dysplasia by downregulating TIGAR. Concomitantly, Rg3 reduced NADP^+^, GSH, and G6PDH levels, thereby depleting antioxidant reserves and enhancing intracellular ROS accumulation (Lv et al., 2022).

Curcumin, a natural phytochemical extracted from the roots of Jianghuang (Turmeric, *Curcuma longa* L.), exhibits antitumor effects (Liu et al., 2023). In GC cells, curcumin micelles amplified ROS production to disrupt redox homeostasis and mitochondrial bioenergetics. By enhancing mitochondrial ROS generation, curcumin impaired OXPHOS and modulated aerobic-condition glycolysis, creating a metabolic crisis that restricted tumor cell growth (Lin et al., 2020). Future research focusing on ROS-sensitive drug delivery systems or combined therapies that synergize ROS induction and glycolytic inhibition will open new avenues for the prevention and treatment of GC.

ROS-related mechanisms require particular attention. Many plant metabolites are redox-active and can induce ROS elevation or scavenging at concentrations that exert non-specific cytotoxicity. Since ROS can secondarily modulate both glycolysis and mitochondrial respiration, changes in ROS levels alone are insufficient to establish specific metabolic target engagement. Therefore, studies should distinguish redox stress from genuine metabolic reprogramming using appropriate antioxidants, mitochondria-specific probes, and flux-based assays, while controlling for the concurrent effects on cell viability and proliferation.

### 5.7 Modulation of the metabolism-immunity axis

The malignant progression of GC is closely associated with immune escape and metabolic abnormalities within the tumor microenvironment (Shang et al., 2024). Abnormal glycolysis in GC cells leads to excessive lactate accumulation, which acidifies the tumor microenvironment and promotes the generation of regulatory T cells (Tregs) (Su et al., 2024). As key immunosuppressive cells, Tregs further facilitate tumor immune escape by inhibiting the anti-tumor activity of effector T cells (Zheng et al., 2023). The combined application of the metabolite derivative Pyrolyzed deketene curcumin (GO-Y022) and the glycolysis inhibitor 2-deoxy-D-glucose (2DG) offers a new direction to break this vicious cycle. Studies have shown that the glycolysis inhibitor 2DG could synergize with GO-Y022 to enhance anticancer effects. On one hand, it inhibited glucose metabolism in tumor cells, reduced the production of ATP and lactate, and induced cell death. On the other hand, it eliminated lactate-mediated promotion of Treg generation to reshape the antitumor immune microenvironment (MaruYama et al., 2023). Through this dual mechanism, GO-Y022 and 2DG provided a new metabolic-immunological dual-modulated strategy for GC treatment.

### 5.8 RTKN

Rho effector protein Rhotekin (RTKN) is significantly upregulated in GC cells and is closely associated with malignant progression (Sun et al., 2019b). RTKN promoted glycolytic flux and sustains intracellular energy supply by enhancing glycolysis-related biological processes, thereby supporting the rapid proliferation and survival of GC cells, indicating its potential value as a key metabolic target (Zheng et al., 2023; Sun et al., 2019b; Wu et al., 2021). Studies have demonstrated that the alcohol extract of Zuojin Pill can suppress cell viability and proliferation in SGC-7901 GC cells, and these inhibitory effects are mediated at least in part by downregulating the expression of RTKN and subsequently attenuating glycolytic activity (Wu et al., 2021). These findings illustrated that the ethanol extract of Zuojin Pill was associated with downregulation of RTKN and attenuation of glycolytic activity in SGC-7901 cells, supporting a possible RTKN-related mechanism rather than definitive RTKN targeting.

## 6 Role of oxidative phosphorylation in gastric precancerous lesions and gastric cancer

**FIGURE 3:**
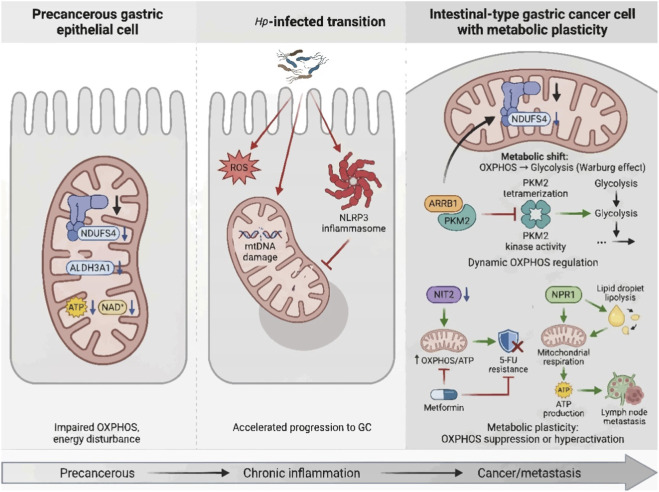
Stage-specific dysregulation of OXPHOS in gastric cancer and its role in metabolic plasticity. This schematic shows the dynamic changes in mitochondrial OXPHOS from precancerous lesions to chronic inflammation and intestinal-type gastric cancer, illustrating how OXPHOS dysregulation drives the Warburg effect, chemoresistance, and metastasis.

Abnormal function of mitochondrial OXPHOS is involved throughout the entire initiation and progression of GC, and its regulatory pattern is characterized by stage-specificity and functional complexity, serving as a core metabolic event in GC metabolic reprogramming (Figure 3). The seemingly contradictory changes in OXPHOS observed in different studies are essentially attributed to its dynamic and plastic regulation.

At the precancerous lesion stage of GC, functional impairment of mitochondrial OXPHOS has already emerged. In typical precancerous lesions including atrophic gastritis, the respiratory chain Complex I exhibited significantly reduced OXPHOS activity. Concomitantly, the expression of Complex I-related proteins (NDUFS4, ALDH3A1) was downregulated, the levels of ATP and NAD^+^ were decreased, and mitochondrial structure was damaged. These findings suggest that energy metabolic disturbance is an early event in GC carcinogenesis (Gruno et al., 2008). At the cancerous stage, OXPHOS reprogramming in GC cells becomes more prominent. Specifically, the expression of OXPHOS Complex I was further reduced in intestinal-type GC tissues, which drove the metabolic switch from OXPHOS to glycolysis and manifested a typical Warburg effect. Notably, no significant differences in this phenotypic alteration were observed in the pure inflammation or precancerous stages (Feichtinger et al., 2017). Aberrant regulation of OXPHOS in GC was also closely associated with the carcinogenic effects of chronic inflammation. Chronic inflammation induced by Hp further promoted energy metabolic reprogramming in gastric mucosal cells and accelerated the progression of GPL by damaging mitochondrial structure, inducing ROS production, and affecting mitochondrial DNA and signaling pathways such as the NLRP3 inflammasome pathway (Toh and Wilson, 2020). As a core process of mitochondrial energy metabolism, OXPHOS not only participated in GC initiation but also exerted complex regulatory effects in shaping the malignant phenotypes of tumors. GC cells achieved dynamic switching of metabolic phenotypes by modulating OXPHOS levels, which explained why elevated OXPHOS has been observed in some studies. This phenomenon was not contradictory to the Warburg effect but represented a metabolic adaptation of cells under different physiological and pathological conditions. For example, β-arrestin 1 (ARRB1) bond to PKM2 and inhibited its tetramerization, reduced kinase activity, and promoted the metabolic shift from OXPHOS to aerobic-condition glycolysis, and high expression of ARRB1 was closely correlated with poor prognosis in GC patients (Yu et al., 2022). Meanwhile, abnormal activation of OXPHOS was also closely linked to malignant processes such as chemoresistance and metastasis in GC. Downregulation of NIT2 enhanced OXPHOS to promote 5-FU resistance in GC cells, whereas OXPHOS inhibitors such as metformin effectively reversed this resistant phenotype and improved chemotherapeutic efficacy (Wang Z. et al., 2024). NPR1 promoted lymph node metastasis of GC by activating lipid droplet lipolysis and mitochondrial OXPHOS, and its overexpression could significantly increase mitochondrial respiratory rate and ATP production (Fu et al., 2025).

Collectively, these studies confirm that abnormalities in the OXPHOS regulatory network are important driving factors for the malignant phenotype of GC and GPL, and modulating OXPHOS-related molecular mechanisms has emerged as a potential new direction for the precise treatment of GC.

## 7 Modulation of oxidative phosphorylation in gastric precancerous lesions and gastric cancer

TCM formulas have shown unique advantages in improving GPL and inhibiting GC progression, with one of their core mechanisms being the repair of mitochondrial functional homeostasis through modulated regulation of the OXPHOS pathway. Specifically, the TCM formula Lianqiao Wendan Decoction was associated with restoration of ATP production, the NAD^+^/NADH ratio, and OXPHOS-related proteins such as NDUFS4 and ALDH3A1 in GPL models (Chi et al., 2025). Another TCM formula, Sijunzi Decoction was associated with changes in OXPHOS-related proteins and metabolites, reduced abnormal glycolytic activation, and improvement of gastric mucosal pathology. The attenuation of its protective effects by OXPHOS inhibitors supports a possible contribution of OXPHOS regulation, although pathway dependence requires further confirmation (Zhu et al., 2024).

In addition, various plant metabolites also exerted anti-GC effects by modulating OXPHOS. For example, curcumin could deplete DNA polymerase γ through the induction of ROS production, reduce mitochondrial DNA content, thereby inhibiting mitochondrial oxygen consumption rate and disrupting the OXPHOS process, and significantly inhibiting GC cell proliferation and blocking *in vivo* tumor growth (Wang L. et al., 2017).

In summary, OXPHOS does not simply increase or decrease during the progression of GC, but presents dynamic characteristics of inhibition at the precancerous stage, dominance of the Warburg effect in the main cancerous stage, and compensatory activation under specific states. This stage-specificity and plasticity constitute the core reason for the differences in conclusions of different studies. Modulating OXPHOS not only provides a new strategy for the precise treatment of GC but also offers an important theoretical basis for explaining the scientific connotation of botanical drugs in the prevention and treatment of GC GPL and malignant progression.

## 8 Clinical challenges and limitations

Numerous plant metabolites have demonstrated potential to modulate glycolytic pathways in the context of GC therapy, with several candidates currently advancing through various phases of clinical trials. A randomized phase IIa study indicated that curcumin in combination with FOLFOX chemotherapy was safe and well-tolerated among patients suffering from metastatic colorectal cancer (Howells et al., 2019). As an adjuvant therapy, nano-curcumin has been shown to be safe and effective in reducing intensive care unit stay, decreasing analgesic use, and improving overall appetite in patients with mild to moderate acute pancreatitis (Chegini et al., 2023). Furthermore, in patients with advanced hepatocellular carcinoma (HCC) and preserved liver function, combination therapy transarterial chemoembolization (TACE) plus ginsenoside Rg3 significantly prolonged overall survival compared to TACE monotherapy (Zhou et al., 2016). Additionally, daily administration of 900 mg berberine combined with mesalazine was well-tolerated in Chinese patients with ulcerative colitis, suggesting a potential synergism that may enhance the anti-inflammatory effects of mesalazine within colonic tissues (Xu et al., 2020).

Despite the potential of botanical drugs in regulating glycolysis in GC cells, current research still has several limitations. First, a significant gap in translational evidence. The vast majority of available studies remain preclinical, with most conducted exclusively in in vitro systems. Direct clinical evidence demonstrating whether botanical drugs or their active metabolites can effectively modulate glycolysis-OXPHOS metabolic plasticity in GC patients, and whether such modulation translates into improved clinical outcomes, remains scarce. Importantly, reductions in tumor-cell glycolysis markers observed in in vitro models cannot be reliably extrapolated to clinical benefit without supportive pharmacokinetic exposure data, validated predictive biomarkers, and rigorously designed controlled clinical trials. For TCM formulas specifically, the lack of standardized clinical dosage forms, inconsistent administration protocols, and unclear correlation between crude drug dose and *in vivo* effective concentration further hinder the generation of high-quality clinical evidence.

Second, the complexity of pharmacodynamic plant metabolites and ambiguity of action mechanisms. For single plant metabolites, synergistic effect of these plant metabolites can achieve multi-modulate anti-tumor effects. For instance, berberine in *C. chinensis* Franch, simultaneously inhibited the HK2 and PI3K/AKT pathways. However, this inherent chemical complexity complicates the precise elucidation of underlying mechanisms. For TCM formulas, the challenge is even more acute: as multi-component preparations containing botanical, animal, and sometimes mineral ingredients, their regulatory effects on glycolysis-OXPHOS are the integrated result of synergistic or antagonistic interactions among hundreds of metabolites. For example, the TCM formula Jianpi Yangzheng Xiaozheng Decoction downregulated HK2 expression by inhibiting the PI3K/AKT/mTOR/HIF-1α cascade, but the specific active plant metabolites such as polysaccharides and flavonoids and their synergistic mechanisms still needed further disassembly and verification. Additionally, most studies on TCM formulas only confirm correlations between the formula and changes in metabolic pathways/proteins, lacking direct evidence such as target binding assays or rescue experiments (e.g., overexpression of AKT or HK2) to verify causal relationships, making it difficult to distinguish whether metabolic modulation is a direct effect or a secondary consequence of overall anti-tumor activity.

Third, pharmacokinetic deficiencies and low bioavailability. The clinical translation of many plant metabolites is impeded by suboptimal pharmacokinetic profiles, including poor water solubility, insufficient oral absorption, and rapid *in vivo* metabolism. For instance, although the Tripterygium extract celastrol inhibited GLUT1/HK2/LDH through multiple targets, its poor water solubility and gastrointestinal absorption led to low oral bioavailability (Freag et al., 2018). Similarly, curcumin exhibited multiple pharmacological effects, such as anti-inflammation and anti-cancer, but it had low bioavailability due to poor water solubility, more than 90% of an oral dose underwent rapid degradation in the alkaline intestinal environment (Rapalli et al., 2020). For TCM formulas, the situation is more complex: the diversity of chemical components leads to inconsistent pharmacokinetic behaviors (e.g., some components are rapidly absorbed while others are poorly soluble), and the lack of systematic pharmacokinetic studies on the whole formula makes it impossible to determine the absorption, distribution, metabolism, and excretion characteristics of key metabolic regulatory components, further limiting clinical translation. To address this issue, the development and application of advanced formulation technologies, such as nanocarriers, should be prioritized. Nanocarriers can encapsulate hydrophobic plant metabolites, improve their solubility, and thus enhance bioavailability.

Fourth, systemic toxicity and the potential for organ damage represent significant concerns. The non-selective biological effects of some plant metabolites may lead to off-target toxicity and damage to normal tissues. For example, matrine has been shown to induce hepatotoxicity in mice via a ROS-dependent mechanism (Liu J. et al., 2020) and to cause nephrotoxicity through a GSK-3β/NRF2-mediated mitochondrial apoptotic pathway (Wang et al., 2023). β-asarone has been identified as promising chemosensitizing agent for GC, but it should be emphasized that this agent is also flagged as carcinogenic with inherent toxicological risks (Uebel et al., 2021). For TCM formulas, the complexity of components increases the risk of potential toxic interactions among ingredients, and the lack of standardized toxicity evaluation systems for multi-component preparations makes it difficult to comprehensively assess safety risks (e.g., long-term administration toxicity, organ-specific toxicity). Consequently, the therapeutic application of such agents necessitates comprehensive and rigorous toxicological evaluation to ensure safety.

## 9 Conclusions and prospects

Aberrant glycolysis is a pivotal metabolic hallmark of GC progression, and multiple enzymes and signaling networks are involved in this process. Preclinical studies suggest that plant metabolites and TCM formulas may modulate glycolysis-related nodes through multi-level mechanisms. For example, DT-13 plus topotecan was associated with reduced HK2 activity and mitochondrial binding, whereas emodin and luteolin were associated with lower HK2 expression and reduced glucose consumption/lactate production. Plant metabolites such as gypenoside and oleanolic acid were reported to modulate Hippo-YAP-related signaling, while ncRNA- and ROS-related mechanisms may provide additional avenues for metabolic intervention. TCM formulas, including Jianpi Yangzheng Xiaozheng Decoction and Xiaotan Sanjie Formula, were associated with inhibition of glycolysis-related phenotypes and increased apoptosis in GC models, together with changes in PI3K/AKT/mTOR/HIF-1α-related markers. However, in most cases the current evidence supports pathway-associated metabolic modulation rather than definitive direct molecular targeting.

Most glycolytic inhibitors remain in preclinical stages, and future efforts should prioritize optimizing combination therapies to overcome tumor metabolic plasticity. Identifying reliable biomarkers to stratify patients for glycolytic modulating is critical. Non-coding RNAs such as miR-21, miR-34a may serve as predictive markers for therapeutic response and drug resistance. The acidic TME driven by lactate promotes immune evasion, necessitating strategies to simultaneously inhibit glycolysis and restore immune function. Combinations of LDHA inhibitors with PD-1 blockers may enhance anti-tumor immunity. Plant metabolites like celastrol and schizandrin A face challenges in bioavailability and toxicity. Nanoparticle-based drug delivery systems or structural modification may improve their therapeutic index. The crosstalk between glycolysis, OXPHOS, and the pentose phosphate pathway presents opportunities for multi-pathway intervention. More focus should be placed on plant metabolites and TCM formulas with dual pathways or mechanisms to increase the standard of care (SOC) treatment sensitivity and reduce therapeutic resistance. Modulating metabolic checkpoints may disrupt energy homeostasis more comprehensively.

In summary, modulating glycolysis in GC holds significant promise, but translating preclinical insights to clinical practice requires interdisciplinary collaboration to address metabolic heterogeneity, TME complexity, and therapeutic resistance. Integrating metabolites-based multi-target strategies with precision medicine approaches may pave the way for innovative GPL and GC therapies.
